# Modelling Negative Feedback Networks for Activating Transcription Factor 3 Predicts a Dominant Role for miRNAs in Immediate Early Gene Regulation

**DOI:** 10.1371/journal.pcbi.1003597

**Published:** 2014-05-08

**Authors:** Marcus J. Tindall, Angela Clerk

**Affiliations:** 1 Department of Mathematics & Statistics, University of Reading, Reading, Berkshire, United Kingdom; 2 School of Biological Sciences, University of Reading, Reading, Berkshire, United Kingdom; University of Tokyo, Japan

## Abstract

Activating transcription factor 3 (Atf3) is rapidly and transiently upregulated in numerous systems, and is associated with various disease states. Atf3 is required for negative feedback regulation of other genes, but is itself subject to negative feedback regulation possibly by autorepression. In cardiomyocytes, Atf3 and Egr1 mRNAs are upregulated via ERK1/2 signalling and Atf3 suppresses Egr1 expression. We previously developed a mathematical model for the Atf3-Egr1 system. Here, we adjusted and extended the model to explore mechanisms of Atf3 feedback regulation. Introduction of an autorepressive loop for Atf3 tuned down its expression and inhibition of Egr1 was lost, demonstrating that negative feedback regulation of Atf3 by Atf3 itself is implausible in this context. Experimentally, signals downstream from ERK1/2 suppress Atf3 expression. Mathematical modelling indicated that this cannot occur by phosphorylation of pre-existing inhibitory transcriptional regulators because the time delay is too short. De novo synthesis of an inhibitory transcription factor (ITF) with a high affinity for the Atf3 promoter could suppress Atf3 expression, but (as with the Atf3 autorepression loop) inhibition of Egr1 was lost. Developing the model to include newly-synthesised miRNAs very efficiently terminated Atf3 protein expression and, with a 4-fold increase in the rate of degradation of mRNA from the mRNA/miRNA complex, profiles for Atf3 mRNA, Atf3 protein and Egr1 mRNA approximated to the experimental data. Combining the ITF model with that of the miRNA did not improve the profiles suggesting that miRNAs are likely to play a dominant role in switching off Atf3 expression post-induction.

## Author Summary

Activating transcription factor 3 (Atf3) is an important regulatory transcription factor which is associated with inflammation, restraint of the immune response and cancer. In this work, we develop a series of mathematical models to understand how Atf3 may be regulated. Informed with data from the literature and our own experiments, we show that self-regulation of Atf3 does not allow for variation between experimentally observed Atf3 mRNA and Atf3 protein expression profiles. A fast-acting signal via phosphorylated RSK is also shown to be implausible for similar reasons. Extending our mathematical model further, we postulate for the first time, that the observed dynamical variation in Atf3 mRNA and protein can be described by microRNAs downstream of RSKs. The further inclusion of an inhibitory transcription factor for Atf3 expression has little effect on these findings.

## Introduction

The concept of immediate early genes (IEGs) was initially established in relation to viral infection of bacterial or eukaryotic cells (e.g. herpesvirus [1]). Here, IEGs are defined as the genes encoding the first phase of mRNAs expressed from the viral genome, relying entirely on pre-existing host proteins. Early and delayed genes are expressed later and require production of new proteins. In mammalian cells, IEGs became defined as those which are regulated by pre-existing transcription factors (TFs) such that increases in IEG mRNA expression are not suppressed by protein synthesis inhibitors (e.g. cycloheximide) [2]. Many IEGs encode transcriptional regulators that are required to modulate downstream gene expression (i.e. second phase genes) and some are inhibitory factors required to terminate transcription. With the discovery of miRNAs that influence mRNA expression and translation [3], [4], some adjustment of the IEG concept is necessary according to whether mRNA regulation is transcriptional or post-transcriptional. Synthesis, production and degradation of miRNAs may be insensitive to inhibitors of protein synthesis as would be any (post-transcriptional) effects on mRNA expression.

Cardiomyocytes, the contractile cells of the heart, withdraw from the cell cycle perinatally. Neonatal rat cardiomyocytes are therefore highly synchronized and, because they do not divide, form a good model for the study of IEG regulation. In these cells, endothelin-1 (ET-1), a Gq protein-coupled receptor (GqPCR) agonist, elicits maximal activation of the entire pool of extracellular signal-regulated kinases 1/2 (ERK1/2, the prototypic mitogen-activated protein kinases) within 3–5 min [5], [6]. The earliest IEGs are substantially and maximally upregulated within 15–30 min, with second phase RNAs being detected within 1 h [7]. ERK1/2 play a major role in the response and promote upregulation of ∼70% of ET-1-responsive transcripts [7], [8]. Recent data indicate that ERK1/2 act together with their downstream substrates p90 ribosomal S6 kinases (RSKs) to regulate RNA expression, with RSKs being required to increase expression of ∼50% of the RNAs upregulated by ET-1 [9]. As in other systems, upregulation of cardiomyocyte IEG mRNAs is acute and transient. For example, activating transcription factor 3 (Atf3) and early growth response 1 (Egr1) mRNAs (each regulated via ERK1/2) are both upregulated maximally by ET-1 within ∼30 min [10]. However, expression of Egr1 mRNA returns to basal by ∼2 h and expression of Atf3 mRNA declines close to basal levels over ∼4 h. This raises the question of negative feedback regulation.

Atf3 is emerging as an extremely important feedback regulator of transcription. Particular emphasis is placed on its role in inflammation and Atf3 is essential for restraining the immune response, but Atf3 is also associated with cancer and cardiac dysfunction [11]–[14]. However, although it is stress-regulated and stress-regulating, Atf3 is upregulated in many systems by growth stimuli including peptide growth factors and GqPCR agonists [7], [15]–[17]. Atf3 forms homo- or heterodimers that bind to ATF/CRE sites in gene promoters to regulate transcription [18]. It is largely regulated at the level of expression, being present at very low levels in quiescent cells and induced as an IEG by a range of extracellular stimuli and cellular stresses. ERK1/2 are particularly implicated in promoting Atf3 mRNA expression and several TFs may be involved [19]–[21]. Atf3 is generally viewed as a transcriptional repressor, particularly when acting as homodimers, and it may repress transcription from its own promoter to limit expression [22]. In cardiomyocytes, Atf3 operates in a negative feedback system with Egr1 and, by binding to the Egr1 promoter, Atf3 inhibits Egr1 transcription [10]. Mathematical modelling of the system demonstrated that this, in itself, could suffice for the transient nature of the Egr1 response. The pivotal role that Atf3 plays in biological systems renders it essential to understand the mechanisms that regulate Atf3 expression. Here, we have developed our original mathematical model to determine whether Atf3 serves as a negative feedback regulator of its own transcription (as suggested [22]) and to explore other mechanisms that may switch off Atf3 mRNA expression. We demonstrate that self-regulation by Atf3 on its own promoter (or, indeed, self-regulation by any transcription factor) is implausible and miRNAs are likely to play a dominant role in switching off Atf3 expression post-induction.

## Results

**Figure 1:**
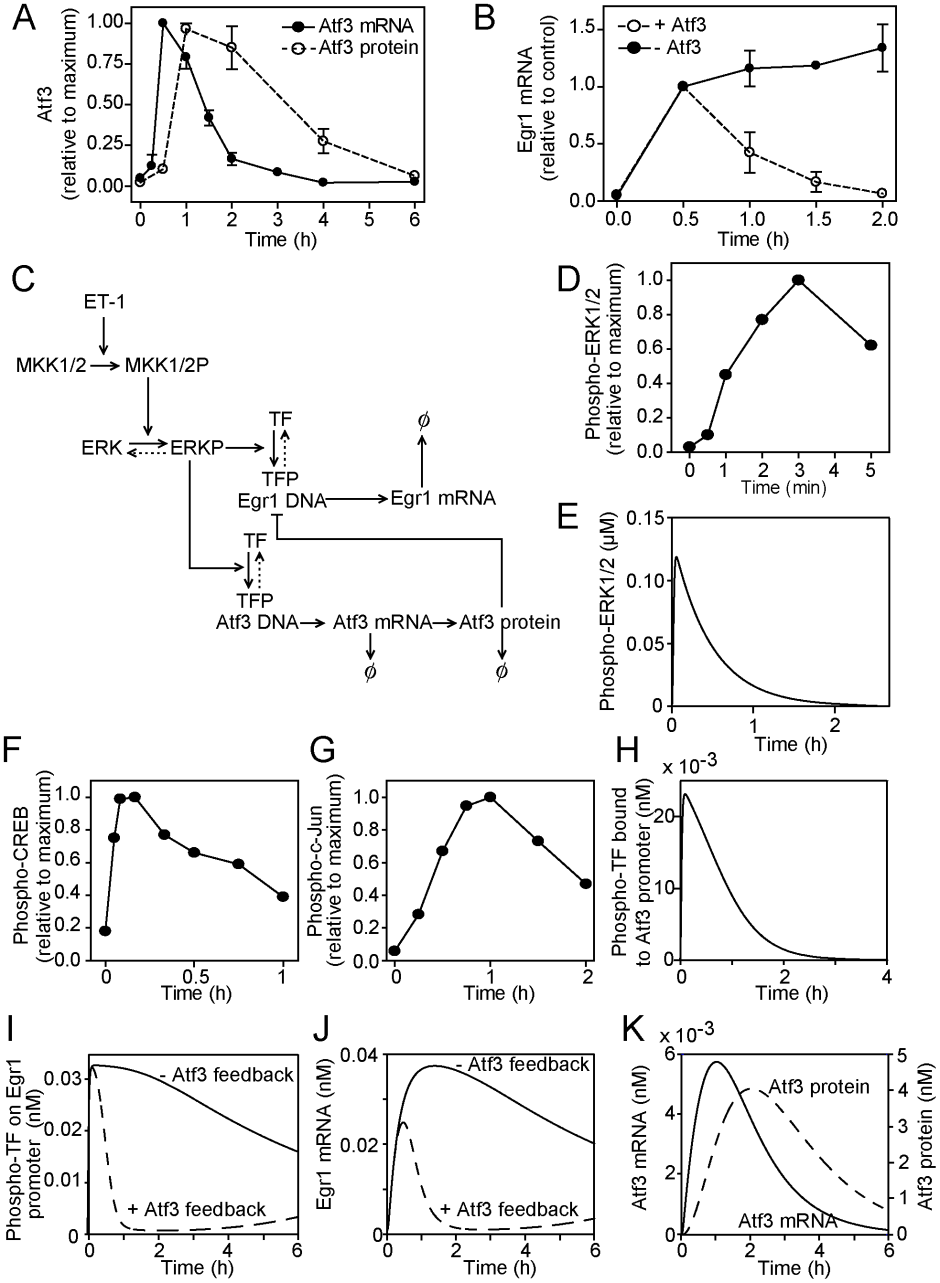
Experimental data for Atf3 and Egr1 regulation in cardiomyocytes exposed to ET-1 and modifications to the original mathematical model for the Atf3/Egr1 negative feedback system. A, Experimental data for Atf3 mRNA (solid circles, solid line) and Atf3 protein (open circle, dashed line) levels in cardiomyocytes exposed to 100 nM ET-1 for the times shown. Results are expressed relative to maximum values as means ± SEM for at least 3 independent experiments (adapted from data in [10]). B, Experimental data for Egr1 mRNA levels in cardiomyocytes exposed to 100 nM ET-1 for the times shown in the presence of endogenous Atf3 (open circles, dashed line) or with inhibition of Atf3 (solid circles, solid line). Results are expressed relative to maximum values as means ± SEM for at least 3 independent experiments (adapted from the data in [10]). C, The original mathematical model [10] was revised (dotted lines) to account for dephosphorylation of ERK1/2 and to include reversible binding of phosphorylated transcription factors to the Egr1 and Atf3 promoters. Lines with arrows indicate protein activation, DNA transcription or protein translation. Blocked lines indicate inhibition. Protein and mRNA degradation is indicated by Ø. D, Experimental data for ERK1/2 phosphorylation in cardiomyocytes exposed to ET-1 for the times shown. Data are means for n = 3 independent experiments and are adapted from the data in [9]. E, Predicted profile from the model for ERK1/2 phosphorylation. F, G, Experimental data for phosphorylation of CREB (F) and c-Jun (G) in cardiomyocytes exposed to ET-1 for the times shown. Data are means for at least 3 independent experiments and are adapted from data in [26], [27]. H, The predicted profile from the model for phosphorylation of the TF bound to the Atf3 promoter was based on experimental data for CREB (F). I, Predicted profile from the model for phosphorylation of the TF bound to the Egr1 promoter was based on experimental data for CREB for the initial increase, but the rate of decreased phosphorylation was adjusted such that, in the absence of Atf3, the profile approximated to that of the experimental data (B). Profiles are shown in the presence (open circle, dashed line) or absence (solid circles, solid line) of Atf3 feedback inhibition. J, Predicted profiles from the model for Egr1 mRNA expression in the presence (open circle, dashed line) or absence (solid circles, solid line) of Atf3 feedback inhibition. K, Predicted profiles from the model for Atf3 mRNA (solid line) or protein (dashed line) expression.

Atf3 mRNA and protein are rapidly and transiently upregulated in cardiomyocytes exposed to ET-1 (Figure 1A) and, since loss of Atf3 produces sustained expression of Egr1 mRNA (Figure 1B), Atf3 negatively regulates Egr1 expression [10]. The initial deterministic ordinary differential equation model generated for this Atf3-Egr1 negative feedback system (detailed in [10]) made the following assumptions: Atf3 and Egr1 are co-regulated through ERK1/2; ERK1/2 increase transactivating activities of TFs pre-bound to each promoter (i.e. the TF/promoter constitutes a single entity regulated by ERK1/2); since ERK1/2 activity remains high for at least 30 min [23], we did not switch off the protein kinase signal. This model predicted that Atf3 alone could suffice to to switch off Egr1 transcription.

To address questions relating to Atf3 transcription we developed the original model further (Figure 1C). Full details of reaction equations, mathematical modelling and additional parameterisation are provided in Text S1. We allowed for the rate of dephosphorylation (i.e. inactivation) of ERK1/2 following stimulation with ET-1, considering that phosphorylation of ERK1/2 is maximal at ∼3 min (Figure 1D, [9]) and declines by ∼90% by 1 h with a return to baseline by 2 h [23]. The modelling profile for ERK1/2 phosphorylation is shown (Figure 1E). We allowed for the rate of phosphorylation and dephosphorylation of TFs bound to each of the Atf3 and Egr1 promoters. In other cells, phosphorylation of CREB and/or c-Jun TFs is implicated in the upregulation of Atf3 transcription [20], [24], [25]. In cardiomyocytes exposed to ET-1, accumulation of phosphorylated (i.e. activated) CREB and c-Jun TFs each requires ERK1/2 signalling [26], [27], making them prime exemplary candidates for the model. Experimentally, maximal phosphorylation of CREB is detected at ∼10 min and declines to ∼25% maximal by ∼60 min (Figure 1F). The time course for phosphorylated c-Jun is delayed (maximal at ∼45–60 min, declining to ∼50% maximal at 2 h (Figure 1G), because of the additional increase in expression of c-Jun protein [26]. ET-1 also increases expression of Myc mRNA (maximal at 1 h) [9] that promotes expression of Atf3 over a longer time frame in cells treated with serum [19], so the signal could be perpetuated over a much longer time period. Here, for the Atf3 promoter, the model was initially developed using the profile for phosphorylated CREB that will give the most rapid rate at which Atf3 transcription is terminated (Figure 1H). The Egr1 promoter is most likely regulated by ternary complexes of Elk1/SRF that can be stimulated either by phosphorylation of Elk1 [28] or an increase in SRF binding [29]. Elk1 is rapidly and transiently phosphorylated in cardiomyocytes exposed to phenylephrine with a similar profile to CREB following exposure to ET-1 [30]. SRF is upregulated by ET-1 with maximal expression of SRF mRNA at ∼1 h [7] and this is likely to perpetuate the response given that our data indicate that, in the absence of Atf3 expression, Egr1 expression is sustained over at least 2 h (Figure 1B). Thus, for the TF regulating Egr1 transcription, we used a similar rate of TF phosphorylation as for CREB with delayed dephosphorylation. Binding of the TF to the Egr1 promoter was competitive with Atf3 and was modelled in the absence or presence of Atf3 (Figure 1I). Egr1 mRNA expression remained sensitive to negative feedback regulation by Atf3 (Figure 1J). With these adjustments, Atf3 mRNA and protein were predicted to decline (Figure 1K), but the profiles were delayed relative to the experimental data (Figure 1A). Thus, merely switching off the signal to promote Atf3 transcription/translation alone cannot account for its subsequent downregulation, indicating that a negative feedback system also operates for Atf3.

### Model extension 1: Atf3 protein inhibits Atf3 transcription

**Figure 2:**
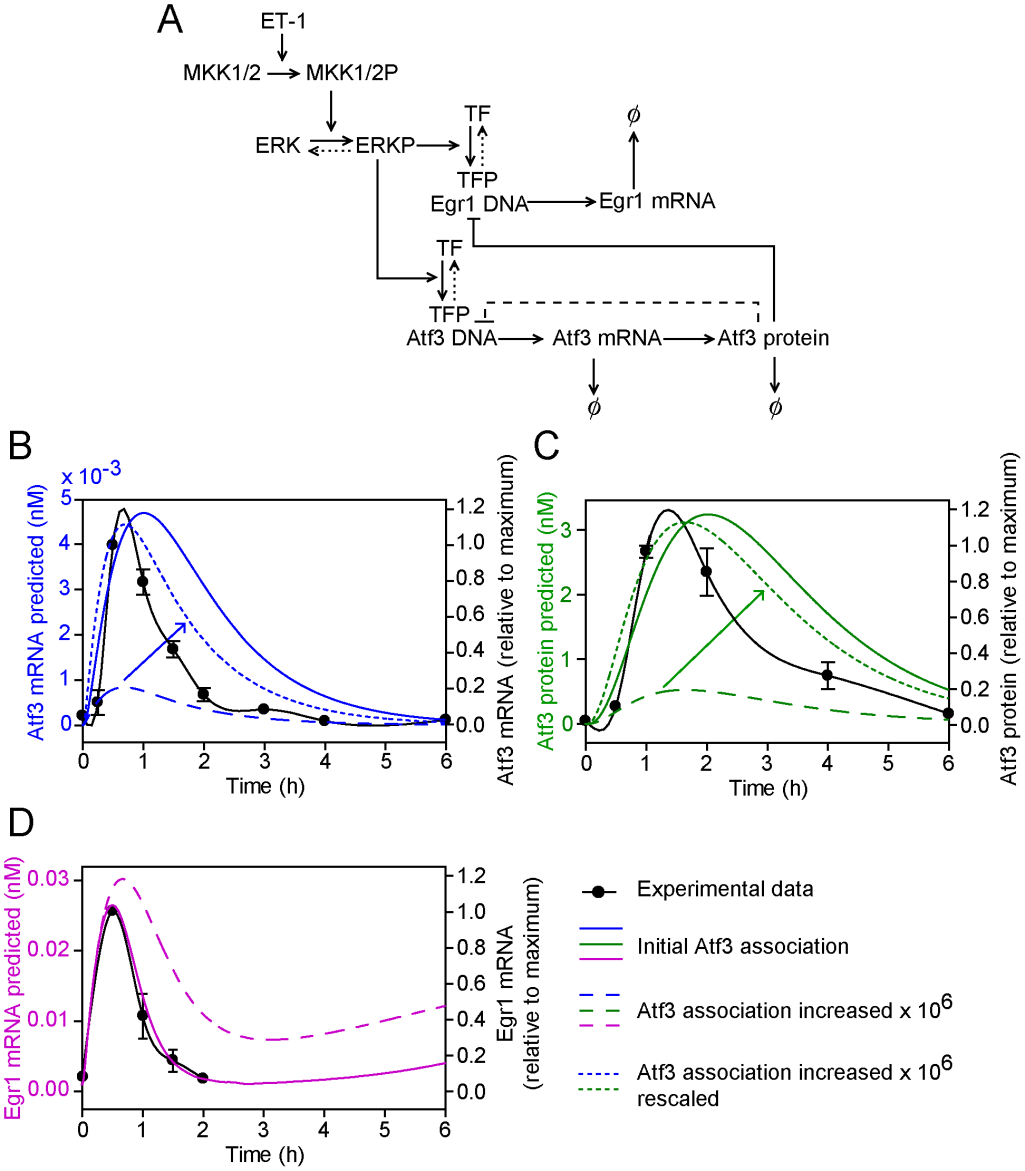
Model extension 1: Atf3 protein inhibits Atf3 transcription in an autorepressive loop. A, The conceptual model of Atf3 self regulation in which Atf3 protein suppresses the transcription of Atf3 (dashed line). Symbols are as for Figure 1C. B, C and D, Atf3 mRNA (B, blue lines), Atf3 protein (C, green lines) and Egr1 mRNA (D, pink lines) concentrations as predicted by the mathematical model using the initial parameter settings (solid lines) and with a 10^6^-fold increase in the association rate of Atf3 for the Atf3 promoter (long dashed lines). In panels B and C, the graphs showing a 10^6^-fold increase in the association rate of Atf3 for the Atf3 promoter were also rescaled for comparison with initial parameter settings (short dashed lines). The predicted curves are superimposed on the experimental data (solid black lines, right axes) to which a spline curve was fitted.

Previous studies used a protein overexpression approach with reporter assays for the Atf3 promoter to provide evidence that Atf3 operates in an autorepression loop and, by binding to an element immediately downstream of the TATA box, it inhibits its own transcription [22]. Since it is difficult to test this experimentally with an endogenous system, we extended the mathematical model developed for the Atf3-Egr1 feedback system to determine whether a direct Atf3 autorepression loop is feasible (Figure 2A). The development of this and subsequent mathematical models is detailed in Text S1. The model extension requires three new parameters. The association constant for Atf3 protein with Atf3 DNA (*K*_11_) was assumed to have an initial value of 0.1 nM. The forward and reverse rates of the Atf3 protein for Atf3 DNA, λ_1_ and λ_−1_, were also required. λ_−1_/λ_1_ was assumed to have a similar value to *K*_11_, but a slow rate of reversal giving a value of λ_1_ = 1×10^5^ (Ms)^−1^. With the initial parameters, there was little effect on Atf3 mRNA, Atf3 protein or Egr1 mRNA profiles (Figure 2, B–D, solid lines) compared with the original model (Figure 1K). Decreasing the disassociation constant of Atf3 protein and Atf3 DNA by 10^6^ produced profiles that were more representative of the experimental data for Atf3 mRNA and protein (Figure 2, B and C, dashed lines), but acute inhibition of Egr1 mRNA expression was lost (Figure 2D, dashed line). Decreasing the ratio of λ_−1_/λ_1_ (informed by a sensitivity analysis) 2 orders of magnitude had no effect on the results. Increasing the ratio 2 orders of magnitude decreased the amount of Atf3 and increased the amount of Egr1 as expected (not shown). We conclude that Atf3 is unlikely to act in an autorepressive manner on its own promoter. If it did, the rate of accumulation of Atf3 mRNA/protein would be reduced such that it could then not significantly influence other gene promoters (in this case Egr1) producing a redundant system. Thus, the Atf3 autorepressive loop is implausible.

### Model extension 2: Simultaneous phosphorylation of an alternative TF by RSKs inhibits Atf3 transcription

**Figure 3:**
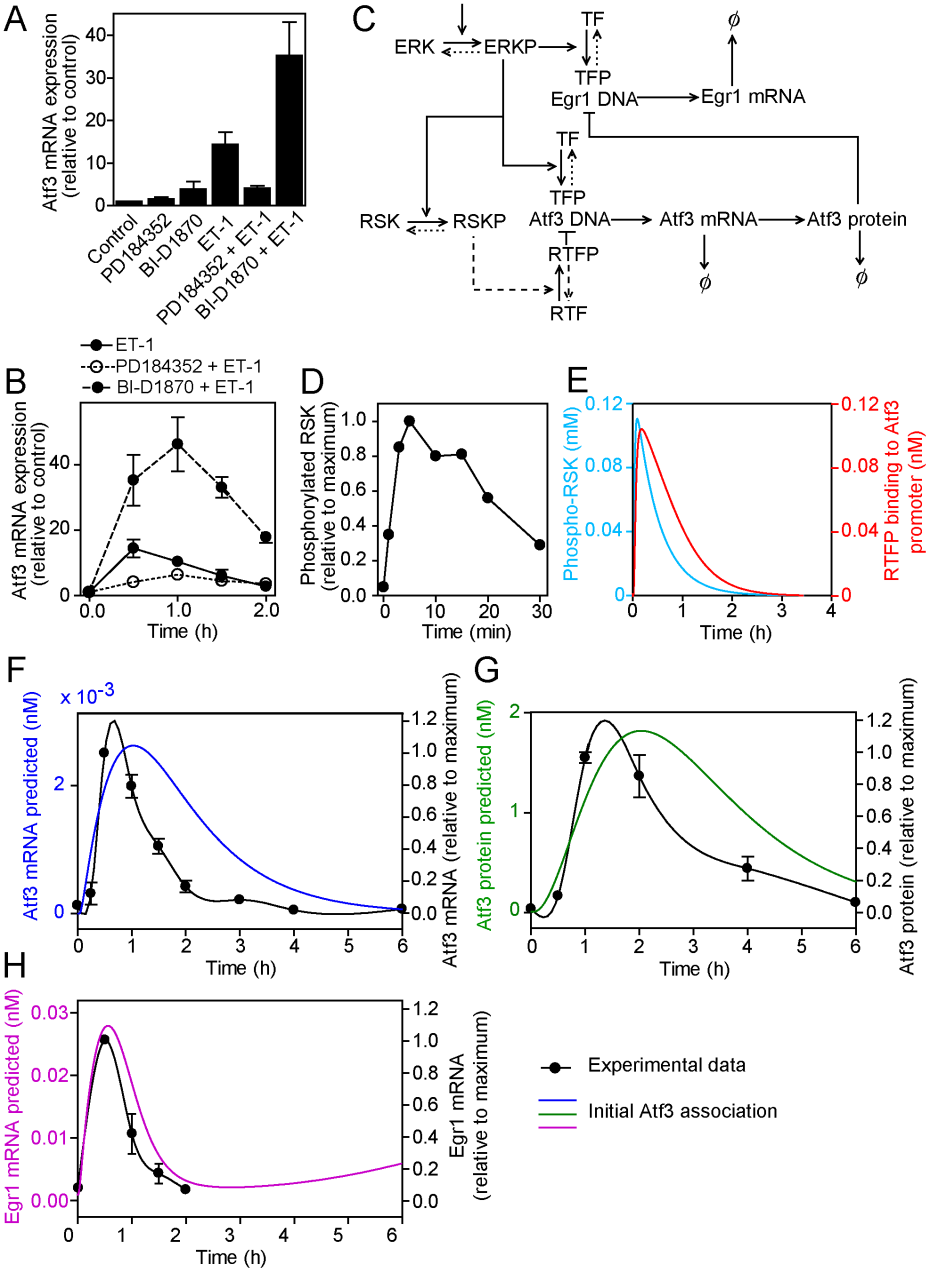
Model extension 2: Simultaneous phosphorylation of an alternative transcription factor by RSKs inhibits Atf3 transcription. A and B, Experimental data for the effects of inhibiting MKK1/2 with PD184352 (2 µM) or inhibiting RSKs with BI-D1870 (10 µM) on Atf3 mRNA expression in unstimulated cells or in cardiomyocytes exposed to ET-1 (100 nM, 1 h; A) or for the times shown (B). Results are expressed relative to controls as means ± SEM for n = 3 (A) or 4 (B) independent myocyte preparations. C, The conceptual model was developed to include phosphorylation of RSKs (RSKP) that phosphorylate a RSK-sensitive TF (RTF) to produce phosphorylated RTF (RTFP) that binds competitively to the Atf3 promoter to suppress Atf3 transcription. Symbols are as for Figure 1C. D, Experimental data for RSK phosphorylation in cardiomyocytes exposed to ET-1 for the times shown. Results are means for n = 3 independent experiments and are adapted from the data in [9]. E, Predicted profiles from the model for RSK phosphorylation (cyan) and RTFP binding to the Atf3 promoter. F, G and H, Atf3 mRNA (F, blue line), Atf3 protein (G, green line) and Egr1 mRNA (H, pink line) concentrations as predicted by the mathematical model using the initial parameter settings The predicted curves are superimposed on the experimental data (solid black lines, right axes) to which a spline curve was fitted.

Exposure of cardiomyocytes to BI-D1870, an inhibitor of RSKs [31], substantially enhances upregulation of Atf3 mRNA by ET-1 (Figure 3A). Although BI-D1870 alone increase ERK1/2 phosphorylation in cardiomyocytes, this over a similar time and to a lesser degree than that induced by ET-1 and it does not affect the activation of ERK1/2 by ET-1 [9]. It is therefore unlikely that the enhancement of Atf3 mRNA expression induced by ET-1 seen in the presence of BI-D1870 is due to its effects on phosphorylation of ERK1/2 and suggests that a negative feedback system downstream of RSKs (or other kinases that may be inhibited by BI-D1870) moderates Atf3 mRNA expression. Interestingly, the effects of BI-D1870 over 2 h (Figure 3B) produced a profile for Atf3 mRNA expression that resembled the predicted profile for Atf3 mRNA in the modifications to the original model (Figure 1K) suggesting that an input from RSKs might suffice to switch off Atf3 transcription. We therefore extended the mathematical model to test the hypothesis that RSKs (as exemplar kinases) phosphorylate nuclear TFs (RTF) to negatively regulate Atf3 transcription (Figure 3C). Experimentally, maximal phosphorylation of RSKs occurs at 5–15 min, declining to ∼25% the maximal level by ∼30 min (Figure 3D); this profile was modelled mathematically (Figure 3E, cyan line; see Text S1 for details). The predicted profile for phospho-RTF binding to the Atf3 promoter is shown in Figure 3E (red line). Because different signals are applied to different proteins, we assumed that the negative signal from phospho-RSK and phospho-RTF was competitive with the positive signal from phospho-ERK1/2 and phospho-TF. New parameters were required for the rate of RSK phosphorylation by phospho-ERK1/2 (*k*_9_), the rate of RSK-P dephosphorylation (*d_10_*), rates of association and disassociation of RTF for Atf3 DNA (*λ*_2_ and *λ*_−2_) and the rates of phosphorylation and dephosphorylation of RTF bound to Atf3 DNA (*k*_10_ and *k*_−10_). A fit-by-eye to the RSK data (Figure 3D) yielded *k*_9_ = 1×10^5^ (Ms)^−1^ and *d_10_* = 5.9×10^−4^ s^−1^. We assumed that *λ*_2_ = *λ*_1_ and *λ*_−2_ = *λ*_−1_, *k*_10_ = *k*_5_ and *k*_−10_ = *k*_−5_, and that the total amount of RSKs is equal to the amount of ERK1/2 (i.e. *R*_0_ = *E*_0_). With the initial parameters, the profiles for Atf3 mRNA and protein remained similar to the original model (Figure 1K), although the amount of each was reduced (Figure 3, F and G) and acute inhibition of Egr1 mRNA remained (Figure 3H). With the competition between phospho-TF and phospho-RTF for Atf3 DNA, increasing the rate of association of phospho-RTF with the Atf3 promoter may produce a profile that approaches that of the experimental data , but acute inhibition of Egr1 mRNA expression becomes lost (not shown). We conclude that the time delay between activation of ERK1/2 relative to RSKs is too small (1–2 min) to result in differential effects on mRNA expression over 0.5–4 h if both act directly on transcription from the same promoter. A greater time delay may be elicited by termination of transcription by an IEG encoding an inhibitory TF (ITF) and/or upregulation of a miRNA (or another factor) promoting Atf3 mRNA degradation and inhibiting Atf3 protein synthesis.

### Model extension 3: Upregulation of an inhibitory TF (ITF) that inhibits Atf3 transcription

**Figure 4:**
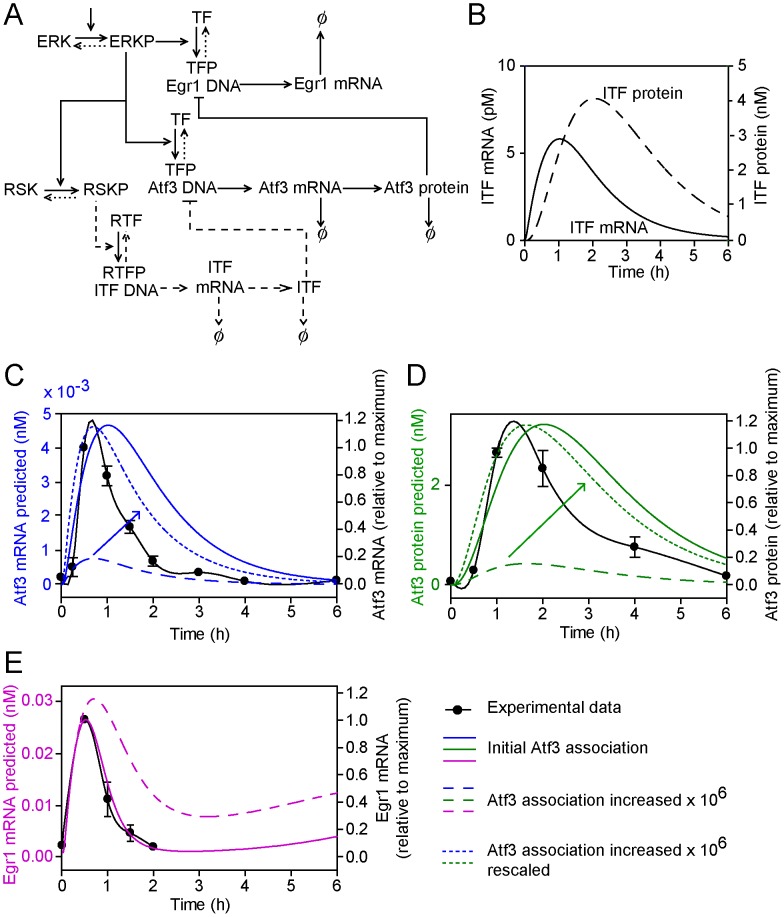
Model extension 3: Upregulation of an ITF that inhibits Atf3 transcription. (A) The conceptual model of the ITF inhibitory pathway for Atf3 transcriptional regulation (dashed lines). Symbols are as for Figure 1C. B, ITF mRNA (left axis, solid line) and ITF protein (right axis, dashed line) concentrations as predicted by the mathematical model. C, D and E, Atf3 mRNA (C, blue lines), Atf3 protein (D, green lines) and Egr1 mRNA (E, pink lines) concentrations as predicted by the mathematical model using the initial parameter settings (solid lines) and with a 10^6^-fold increase in the association rate of ITF for the Atf3 promoter (long dashed lines). In panels C and D, the graphs showing a 10^6^-fold increase in the association rate of ITF for the Atf3 promoter were also rescaled for comparison with initial parameter settings (short dashed lines). The predicted curves are superimposed on the experimental data (solid black lines, right axes) to which a spline curve was fitted.

We next tested the hypothesis that downregulation of Atf3 mRNA expression (we retained the assumption that this is via RSKs) is mediated by upregulation of an ITF (Figure 4A), making the assumption that the negative effect of ITF was competitive with the positive signal from ERKs. Five further parameters are required for this model: the disassociation constant of ITF protein for Atf3 DNA (*K*_15_), the rate of ITF transcription (*k*_13_), the rate of translation of ITF protein (*k*_14_), ITF mRNA degradation (*d*_4_), and the rate of ITF protein degradation (*d*_5_). On the basis that ITF is an IEG, we assumed that the disassociation constant of ITF protein was equal to that of TF for Atf3 DNA (*K*_15_ = *K*_10_), rates of transcription and translation of ITF are similar to those for Atf3 (i.e. *k_13_* = *k*_6_ and *k*_14_ = *k*_8_), and the rates of degradation of each mRNA and protein are similar (i.e. *d*_4_ = *d*_2_ and *d*_5_ = *d*_3_) producing the profiles for ITF mRNA and protein shown in Figure 4B. With initial conditions in which ITF binding to Atf3 DNA is similar to Atf3 binding to the Egr1 promoter (Figure 4, C and D, solid lines), there was little change in the profiles compared with the original model (Figure 1K). Increasing the rate of binding of ITF to Atf3 DNA by 10^6^ produced profiles that were more similar to the experimental data (Figure 4, C and D, dashed lines), but inhibition of Egr1 transcription was reduced (Figure 4E, dashed line).

### Model extension 4: Upregulation of one or more miRNAs that regulate Atf3 mRNA and/or protein expression

**Figure 5:**
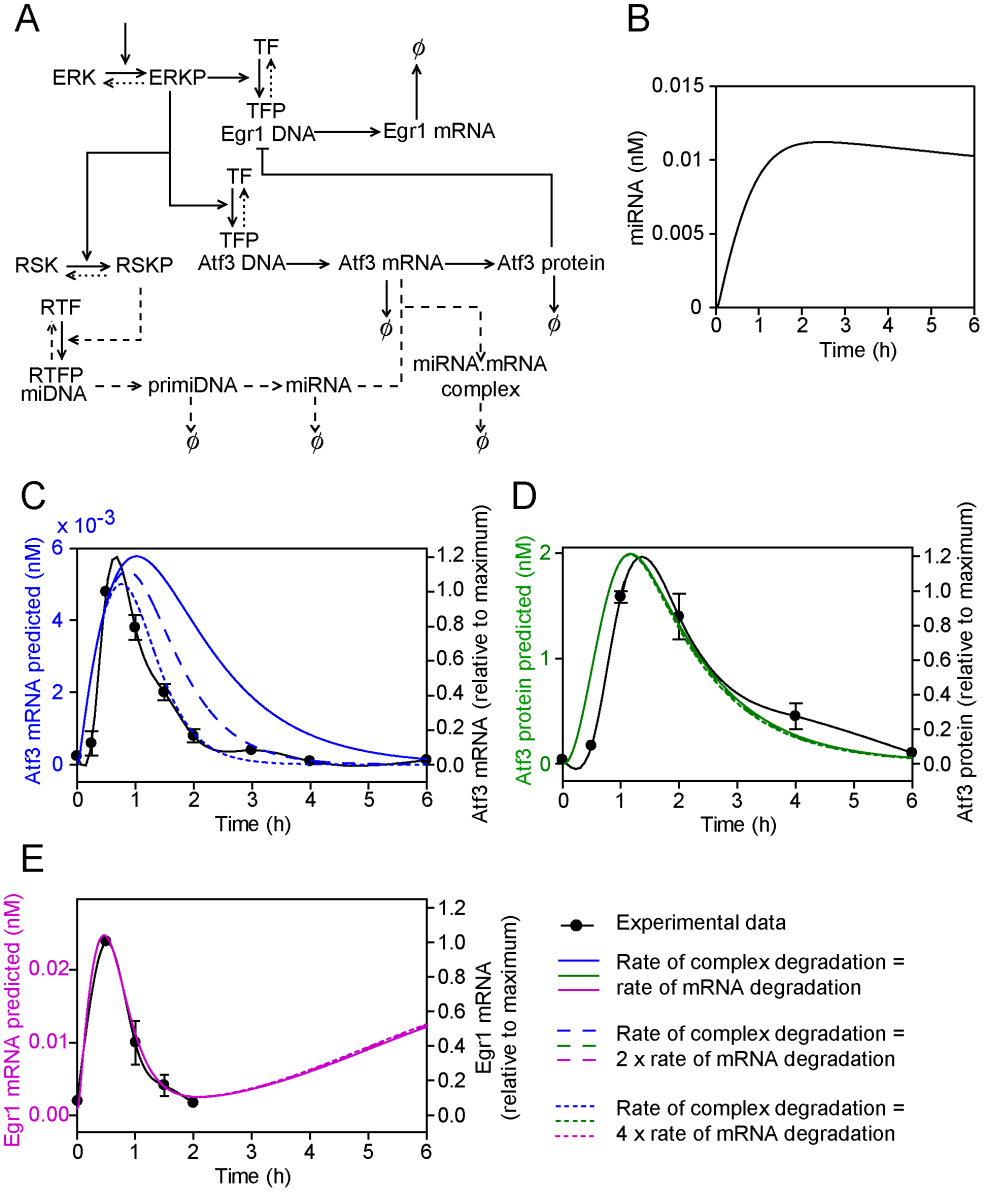
Model extension 4: Upregulation of one or more miRNAs inhibits Atf3 mRNA and/or protein. A, The conceptual model of the miRNA inhibitory pathway for Atf3 transcriptional regulation. Symbols are as for Figure 1C. B, The predicted dynamical variation in miRNA concentration. C, D and E, Predicted concentrations of total Atf3 mRNA (i.e. mRNA + miRNA.mRNA complex) (C, blue lines), Atf3 protein (D, green lines) and Egr1 mRNA (E, pink lines) when the rate of degradation of Atf3 mRNA within the miRNA.mRNA complex is the same as for degradation of free Atf3 mRNA (solid lines), is increased 2-fold (long dashed lines) or is increased 4-fold (short dashed lines). The predicted curves are superimposed on the experimental data (solid black lines, right axes) to which a spline curve was fitted.

Transcriptomics studies using Affymetrix exon arrays [9] identified several miRNAs that are regulated in cardiomyocytes by ET-1 within 1 h and whose upregulation is inhibited by BI-D1870 (Table 1; since we have not performed miRNA profiling of cardiomyocytes, this is unlikely to be a full representation of miRNAs in this category). Atf3 expression may be regulated by miRNAs (e.g. Mir663 suppresses Atf3 mRNA expression in endothelial cells subjected to shear stress [32]). We therefore tested the hypothesis that upregulation of one or more miRNAs (we retained the assumption that this is downstream of RSKs as exemplar kinases) is required to downregulate Atf3 mRNA expression (Figure 5A). This extension to the model introduces eight new parameters. With little/no published information on rates of miRNA turnover, we informed the model as follows. The rate of phosphorylation/dephosphorylation for RTF binding to miDNA was assumed to be equal to the rate of phosphorylation/dephosphorylation of TFs bound to Egr1 and Atf3 DNA by ERK-P (*k*_16_ = *k*_3_; *k*_−16_ = *k*_−3_). The rate of miDNA transcription was assumed to be the same as that of Atf3 transcription (*k*_17_ = *k*_6_) and the rate at which premiRNA is converted to miRNA (*k*_18_) was assumed to be double the rate of Atf3 translation (0.5/s). The rate at which miRNA associates with Atf3 mRNA (*k*_19_) was assumed to be equivalent to the rate set for Atf3 protein binding to Egr1 DNA (1×10^5^ (Ms)^−1^) and the reverse rate (*k*_−19_) was set at 5×10^−5^/s, the value that gave the best qualitative fit to the experimental data. The rates of decay of premiRNA (*d*_6_) and miRNA (*d*_7_) were assumed to be ∼24 h in line with recently published data [33], [34]. The rate of decay of the complex miRNA*·*mRNAAtf3 (*d*_8_) was initially assumed to be the same as that of Atf3 mRNA (*d*_8_ = *d*_3_). Mathematical modelling for the production of miRNA is shown in Figure 5B.

**Table 1:** miRNAs upregulated in cardiomyocytes by ET-1 via ERK1/2 and RSKs.

Probeset	Gene symbol	Raw values	Control	PD184352	BI-D1870	ET-1	PD184352 + ET-1	BI-D1870 + ET-1
7070060	Mir193	118	1.00	0.993	0.642	2.329	1.370	0.760
7135292	Mir19b-1	41	1.00	0.845	0.901	1.506	1.043	0.751
7373217	Mir222	37	1.00	1.039	1.007	2.970	0.979	1.603
7288447	Mir31	102	1.00	0.680	0.602	2.354	1.140	0.827

Cardiomyocytes were unstimulated (Control), exposed for 1 h to 2 µM PD184352 (to inhibit ERK1/2 signaling), 10 µM BI-D1870 (to inhibit RSKs) or 100 nM ET-1 alone, or exposed to ET-1 in the presence of each inhibitor. Results are means (n = 3) expressed relative to controls. Data were taken from [9], [10].

miRNAs may increase mRNA degradation (within a complex with Ago2) or silence mRNA expression (within Ago1, Ago3 or Ago4). Computational modelling has been used to predict/confirm that the increase in mRNA degradation induced by miRNAs lies within the range 1.3- to 6.4-fold [35]. We first assumed that potential miRNA(s) had a negligible effect on the rate of degradation (i.e. the rate of degradation of the miRNA/mRNA complex was similar to the free mRNA). In this case, the total Atf3 mRNA (i.e. free Atf3 mRNA + miRNA/mRNA complex) dynamical profile did not match the experimental data (Figure 5C, solid line). However, since the miRNA/mRNA complex is assumed to be translationally incompetent, the protein profile was significantly affected and approximated to the experimental profile (Figure 5D, solid line). If we assumed that potential miRNA(s) increased the rate of degradation of Atf3 mRNA from the miRNA/mRNA complex 2-fold, the total Atf3 mRNA profile approached that of the experimental data (Figure 5C, long dashed line). Increasing the rate of degradation 4-fold gave a profile that approximated to the experimental data (Figure 5C, short dashed line). The protein profile was relatively unaffected by increasing the rate of degradation of the miRNA/mRNA complex (Figure 5D), indicating that miRNAs have a dominant effect on protein expression irrespective of their effect on mRNA expression. In all cases, the predicted Egr1 mRNA profile approximated to the experimental data over the 2 h period studied (Figure 5E), although subsequently Egr1 mRNA levels were predicted to increase as Atf3 protein is lost.

### Combining model extensions 3 and 4: Upregulation of both an ITF and miRNA(s) is required for inhibition of Atf3 mRNA and protein

**Figure 6:**
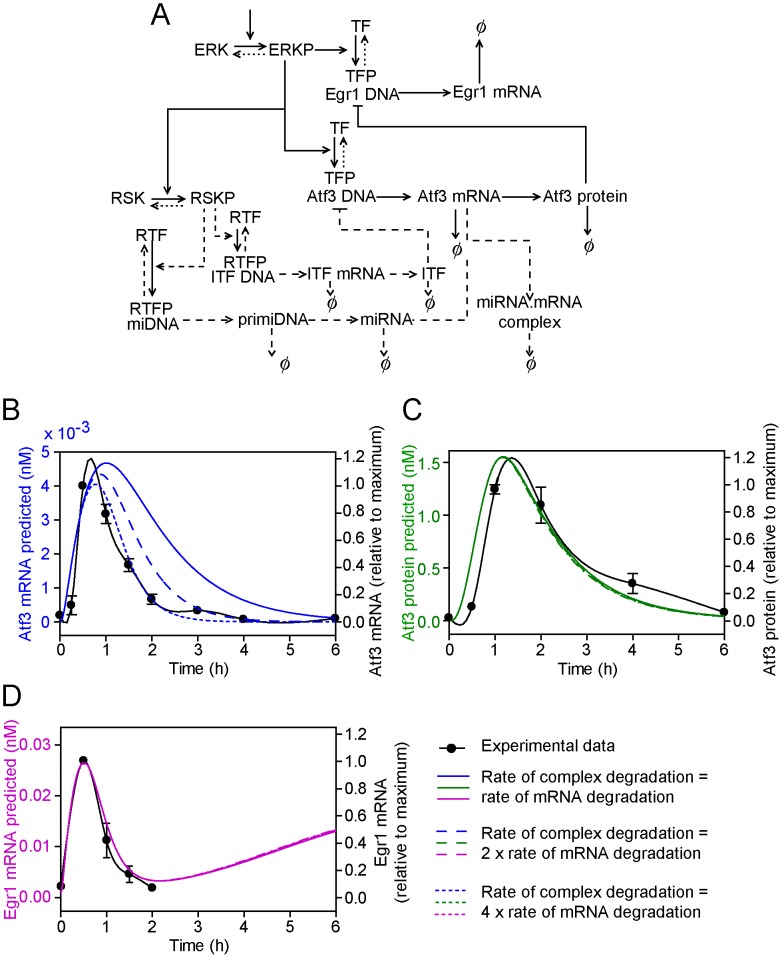
Combined model extensions 3 and 4: Upregulation of both an ITF and miRNA(s) is required for inhibition of Atf3 mRNA and protein. (A) The conceptual model of the ITF and miRNA inhibitory pathways for Atf3 transcriptional regulation (dashed lines). Symbols are as for Figure 1C. B, C and D, Predicted concentrations of total Atf3 mRNA (i.e. mRNA + miRNA.mRNA complex) (B, blue lines), Atf3 protein (C, green lines) and Egr1 mRNA (D, pink lines) when the rate of degradation of Atf3 mRNA within the miRNA.mRNA complex is the same as for degradation of free Atf3 mRNA (solid line), is increased 2-fold (long dashed lines) or is increased 4-fold (short dashed line). The predicted curves are superimposed on the experimental data (solid black lines, right axes) to which a spline curve was fitted.

We combined the two systems for ITF and miRNA inhibition of Atf3 to investigate whether an ITF may influence the profiles in the presence of miRNA(s) (Figure 6A). Profiles for total Atf3 mRNA (Figure 6B), Atf3 protein (Figure 6C) and Egr1 mRNA (Figure 6D) were similar to those for model extension 4 (Figure 5, C–E) indicating that miRNAs alone could suffice to switch off Atf3 mRNA and protein expression.

## Discussion

In considering mechanisms of regulation of IEG expression, the emphasis is usually on the signals and transcriptional regulators required for upregulation. Here, we explored possible mechanisms associated with downregulation of IEG expression and termination of the response. We focused on Atf3 as an IEG in which there is great interest because of its association with inflammation, cancer and cardiac dysfunction [11]–[14].

The report that Atf3 can bind to its own promoter to inhibit transcription [22] is, at first sight, attractive. The experimental approaches used remain the most appropriate, but all such studies are potentially subject to artefacts resulting from overexpression or elimination of a single transcription factor in isolation. Developing a mathematical model provides an alternative means of testing the hypothesis. In this case, the model demonstrated that such a negative feedback loop is implausible in the context of our cardiomyocyte system (Figure 2). This raises the question of whether any such self-regulating transcriptional system can operate. For an inhibitory TF, the model prediction is that a redundant feedback loop is most likely to be generated in which the TF serves to regulate only its own expression, restricting the possibility that it accumulates to a sufficient degree to operate in an efficient inhibitory loop with another gene. The scenario would differ in the case of a positive TF driving expression of second phase genes. Here, self-regulation can provide a means of moderating downstream gene expression.

Our experimental data suggest that, whereas ERK1/2 themselves promote upregulation of Atf3, downstream activation of RSKs (or other BI-D1870-sensitive kinases) is potentially important in terminating Atf3 mRNA and, presumably, protein expression (Figure 3, A and B). This resulted in the development of three further extensions to the model. Whilst we have included biologically appropriate mechanisms at each model revision one outcome of this has been an increase in the signalling cascade complexity. This in itself has led to a more robust signalling response.

The initial concept, that delayed activation of RSKs may result in delayed activation of a pre-existing inhibitory TF for signal termination, appeared attractive. However, model extension 2 demonstrated that direct activation of the putative RSK-responsive RTF was almost simultaneous with activation of the ERK1/2-responsive TF and simply titrated down the levels of Atf3 mRNA rather than altering the profile. Thus, the delay between activation of ERK1/2 and activation of RSKs is insufficient for this scenario to be viable and a greater delay is required before initiation of the negative signal. Interestingly, since a delay is necessary, this also negates the possibility of other acute events (e.g. histone deacetylation) being responsible for terminating Atf3 mRNA expression. The timing strongly suggests that a second transcriptional event is required, either to produce an alternative inhibitory TF (termed ITF, model extension 3) and/or one or more miRNAs (model extension 4). Here, developing first model extensions 3 and 4 independently and then combining the two strongly suggested that an ITF would not affect Atf3 mRNA and protein expression whereas miRNA(s) most probably would suffice to generate the profiles seen experimentally. Interestingly, the miRNA effect was particularly dominant on the Atf3 protein profile. The model also demonstrated that a modest increase in the rate of degradation of Atf3 mRNA by the miRNA within the range predicted by other computational models [35], [36] could suffice for the Atf3 mRNA profiles to approximate to the experimental data. Further studies of cardiomyocyte miRNAs are clearly required.

## Materials and Methods

### Quantitative PCR and microarray data

Neonatal rat cardiomyocytes were prepared as previously described [37] and exposed to 2 µM PD184352 (to inhibit the ERK1/2 cascade [38]) or 10 µM BI-D1870 (to inhibit RSKs [31]) for 70 min, to 100 nM ET-1 for 1 h or to inhibitor for 10 min prior to addition of ET-1 (1 h). PD184352 and BI-D1870 were from Enzo Life Sciences and were dissolved in DMSO. ET-1 was from Bachem UK. RNA was extracted and quantitative PCR performed as described in [10]. Affymetrix microarray data for cardiomyocytes exposed to ET-1 in the absence or presence of PD184352 or BI-D1870 were published in [9]. The data are available from ArrayExpress (accession nos. E-MIMR-3, E-MIMR-37, E-MEXP-3393, E-MEXP-3394, E-MEXP-3678, E-MEXP-3679). Probeset sequences that were significantly upregulated and not assigned to a protein-coding gene were analysed by BLAST search against the rat and mouse genomes (www.ncbi.nlm.nih.gov/genome/seq/BlastGen/BlastGen.cgi?taxid=10116; www.ncbi.nlm.nih.gov/genome/seq/BlastGen/BlastGen.cgi?taxid=10090; cross-species megaBLAST) to identify those which recognise miRNAs.

Data analysis and curve-fitting to experimental data used GraphPad Prism.

### Mathematical modelling

Full details of the mathematical modelling are provided in Text S1.

#### Sensitivity analysis

A thorough local sensitivity analysis of each of the mathematical models was undertaken in which each parameter value was varied 10- and 100-fold above and below its originally determined value in fitting to the experimental data (phosphorylated ERK, phosphorylated RSK, phosphorylated CREB, phosphorylated c-Jun, Atf3 mRNA, Atf3 and Egr1 mRNA). Varying the parameter values associated with the extension of our original mathematical model whereby phosphorylation of transcription factors for Atf3 and Egr1 were explicitly included, did not give any unexpected results, for instance variation in the rate of MKK1/2 or ERK phosphorylation did not affect the fit to the data. The fit to Egr1 mRNA data was found to be most sensitive to those parameters which decrease the amount of available Atf3 (decreasing Atf3 transcription, decreasing Atf3 translation, increasing Atf3 mRNA and Atf3 degradation) and the rate at which Atf3 supresses Egr1 mRNA.

A sensitivity analysis of the two models in which either Atf3 protein or RSKs inhibit Atf3 transcription showed that no increase or decrease in parameters associated with each model improved the fits shown in Figures 2 and 3, respectively. The fit of each model was particularly sensitive to how much Atf3 was produced (for instance increasing the rate of RTF association for Atf3 DNA 10-fold simply decreases the amount of Atf3 such that the amount of Egr1 mRNA increases, removing the transiently observed decrease around 2 h).

We found that the fits between the remaining three models (ITF inhibition of Atf3, miRNA regulation of Atf3 mRNA and a combination of the two) and the experimental data were particularly robust to variations in parameters associated with them. For instance increasing the rate of phosphorylation or reverse phosphorylation of the ITF bound transcription factor 100-fold or any of the degradation rates associated with ITF mRNA or protein did not alter the transiently observed behaviour of the model solutions; any increase or decrease in ITF simply affected the Atf3 concentration. Similar results were found for the miRNA model extension and the combined extension of ITF and miRNA upregulation.

## Supporting Information

Text S1 Details of the mathematical models including their derivation, parameterisation, sensitivity analysis and further solution details.
(PDF)
