# Supplementary material for: Modelling Negative Feedback Networks for Activating Transcription Factor 3 Predicts a Dominant Role for miRNAs in Immediate Early Gene Regulation
Source: PLoS Comput Biol. 2014 May 8;10(5):e1003597. doi: 10.1371/journal.pcbi.1003597 (PMC4014390; doi:10.1371/journal.pcbi.1003597)
Supplement: Text S1 — Details of the mathematical models including their derivation, parameterisation, sensitivity analysis and further solution details. (PDF) [file pcbi.1003597.s001.pdf]

Modelling negative feedback networks for activating transcription factor 3 predicts a dominant role for miRNAs in immediate early gene regulation

M.J. Tindall and A. Clerk

Text S1

We detail each of the mathematical models developed to consider the possible pathway interactions shown in Figure S1 in Text S1. All of the mathematical models were solved using the inbuilt stiff differential equation solver ode15s in Matlab (Mathworks, Version 7.11.0.584).

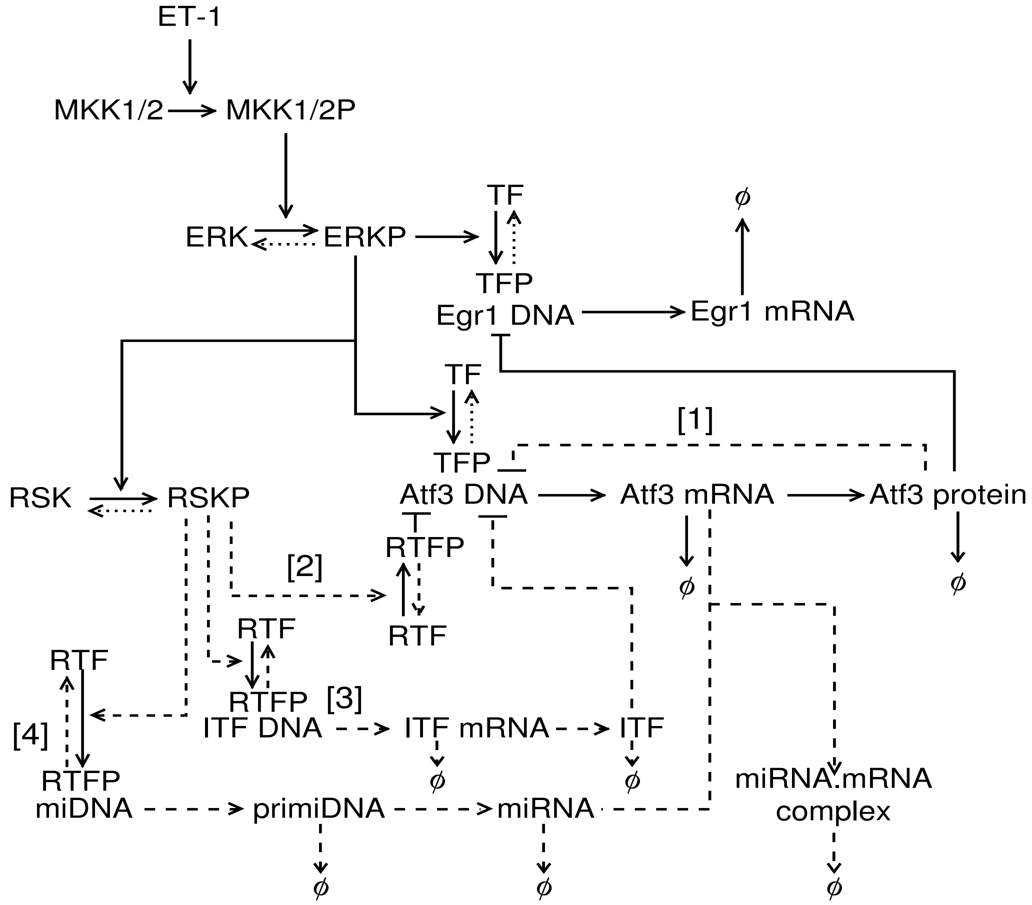

Figure S1: **Potential feedback regulation of Atf3 and Egr1 mRNA expression**. Known pathways (solid lines) and 4 hypothetical pathways (dotted lines) controlling the expression of Atf3 mRNA are shown. Hypothetical pathways are numbered [1]-[4]. Arrows indicate protein activation/upregulation, mRNA transcription or protein translation whilst negative regulation is shown by horizontal lines. The symbol "ϕ" indicates degradation of the respective mRNA/protein/complex as indicated.

S1 Revised original model: Atf3 protein inhibits transcription of Egr1

In the original model in which Atf3 regulates Egr1 mRNA (S6) the action of phosphorylation of the transcription factors driving Egr1 and Atf3 mRNA expression levels were implicitly assumed. In this model revision we explicitly assume the binding of transcription factors to the associated Egr1 and Atf3 genes and their subsequent phosphorylation by phosphorylated ERK. This allows us to include a description of phosphorylated ERK de-phosphorylation which was not considered in our earlier work.

S1.1 Reaction equations

The phosphorylation of MKK by ET-1 is denoted by

$$\text{ET-1} + \text{MKK} \xrightarrow{k_1} \text{ET-1} + \text{MKK-P}, \tag{1}$$

which subsequently phosphorylates the unphosphorylated ERK

$$\text{MKK-P} + \text{ERK} \xrightarrow{k_2} \text{MKK-P} + \text{ERK-P}. \tag{2}$$

ERK-P subsequently de-phosphorylates such that

$$\text{ERK-P} \xrightarrow{d_9} \phi. \tag{3}$$

ERK-P is now free to phosphorylate transcription factors of Egr1 (TF_{Egr1}) and Atf3 DNA (TF_{Atf3}), which we assume are already bound to the DNA of each species, such that

$$\text{TF}_{\text{Egr1}} \cdot \text{DNA}_{\text{Egr1}} + \text{ERK-P} \underset{k_{-3}}{\overset{k_3}{\rightleftharpoons}} \text{TF-P}_{\text{Egr1}} \cdot \text{DNA}_{\text{Egr1}} + \text{ERK-P} \quad , \tag{4}$$

and

$$\text{TF}_{\text{Atf3}} \cdot \text{DNA}_{\text{Atf3}} + \text{ERK-P} \underset{k_{-5}}{\overset{k_5}{\rightleftharpoons}} \text{TF-P}_{\text{Atf3}} \cdot \text{DNA}_{\text{Atf3}} + \text{ERK-P}, \tag{5}$$

which are subsequently transcribed to produce the associated mRNAs

$$\text{TF-P}_{\text{Egr1}} \cdot \text{DNA}_{\text{Egr1}} \xrightarrow{k_4} \text{mRNA}_{\text{Egr1}}, \tag{6}$$

and

$$\text{TF-P}_{\text{Atf3}} \cdot \text{DNA}_{\text{Atf3}} \xrightarrow{k_6} \text{mRNA}_{\text{Atf3}}, \tag{7}$$

Both the phosphorylated bound transcription factors dephosphorylate in time

$$\text{TF-P}_{\text{Egr1}} \cdot \text{DNA}_{\text{Egr1}} \overset{d_{p1}}{\to} \text{TF}_{\text{Egr1}} \cdot \text{DNA}_{\text{Egr1}} \tag{8}$$

$$\text{TF-P}_{\text{Atf3}} \cdot \text{DNA}_{\text{Atf3}} \overset{d_{p2}}{\to} \text{TF}_{\text{Atf3}} \cdot \text{DNA}_{\text{Atf3}} \tag{9}$$

and the mRNAs subsequently degrade

$$\text{mRNA}_{\text{Egr1}} \overset{d_1}{\to} \phi, \qquad \text{mRNA}_{\text{Atf3}} \overset{d_2}{\to} \phi. \tag{10}$$

Here $\cdot$ denotes a complex and ϕ the degraded entity.

The suppression of Egr1 mRNA transcription by Atf3 is described by

$$\text{TF-P}_{\text{Egr1}} \cdot \text{DNA}_{\text{Egr1}} + \text{Atf3} \quad \underset{k_{-7}}{\overset{k_7}{\rightleftharpoons}} \quad \text{TF-P}_{\text{Egr1}} \cdot \text{DNA}_{\text{Egr1}} \cdot \text{Atf3}. \tag{11}$$

Finally the translation of Atf3 mRNA to Atf3 protein and subsequent degradation of the protein are denoted by

$$\text{mRNA}_{\text{Atf3}} \overset{k_8}{\to} \text{Atf3} \quad \text{and} \quad \text{Atf3} \overset{d_3}{\to} \phi_P, \tag{12}$$

respectively, where ϕ_P denotes degraded protein. In this work we do not explicitly account for the degraded mRNAs, Atf3 protein or dephosphorylated ERK-P.

S1.2 Mathematical Model

Applying the Law of Mass Action (S8) to equations (1)-(12) yields

$$\frac{dm}{dt} = -k_1 e_T m, \tag{13}$$
$$\frac{dm_P}{dt} = k_1 e_T m, \tag{14}$$
$$\frac{dE}{dt} = -k_2 m_P E, \tag{15}$$
$$\frac{dE_P}{dt} = k_2 m_P E - d_9 E_P, \tag{16}$$
$$\frac{dT_E}{dt} = -k_3 E_P T_E + k_{-3} E_P T_{EP} + d_{p1} T_{EP}, \tag{17}$$
$$\frac{dT_{EP}}{dt} = k_3 E_P T_E - k_{-3} E_P T_{EP} - k_7 T_{EP} A + k_{-7} S - d_{p1} T_{EP}, \tag{18}$$
$$\frac{dT_A}{dt} = -k_5 E_P T_A + k_{-5} T_{AP} E_P + d_{p2} T_{AP}, \tag{19}$$
$$\frac{dT_{AP}}{dt} = k_5 E_P T_A - k_{-5} T_{AP} E_P - d_{p2} T_{AP}, \tag{20}$$
$$\frac{dM_E}{dt} = k_4 T_{EP} - d_1 M_E, \tag{21}$$
$$\frac{dM_A}{dt} = k_6 T_{AP} - d_2 M_A, \tag{22}$$
$$\frac{dA}{dt} = k_8 M_A - k_7 T_{EP} A + k_{-7} S - d_3 A, \tag{23}$$
$$\frac{dS}{dt} = k_7 T_{EP} A - k_{-7} S, \tag{24}$$

where e_T represents the concentration of ET-1 (denoted e_T=[ET-1] and assumed constant), m=[MKK], m_P=[MKK-P], E=[ERK], E_P=[ERK-P], T_E=[$\mathrm{TF}_{\mathrm{Egr1}} \cdot \mathrm{DNA}_{\mathrm{Egr1}}$], T_{EP}=[$\mathrm{TF\text{-}P}_{\mathrm{Egr1}} \cdot \mathrm{DNA}_{\mathrm{Egr1}}$], T_A=[$\mathrm{TF}_{\mathrm{Atf3}} \cdot \mathrm{DNA}_{\mathrm{Atf3}}$], T_{AP}=[$\mathrm{TF\text{-}P}_{\mathrm{Atf3}} \cdot \mathrm{DNA}_{\mathrm{Atf3}}$], M_E=[$\mathrm{mRNA}_{\mathrm{Egr1}}$], M_A=[$\mathrm{mRNA}_{\mathrm{Atf3}}$], S=[$\mathrm{TF\text{-}P}_{\mathrm{Egr1}} \cdot \mathrm{DNA}_{\mathrm{Egr1}} \cdot \mathrm{Atf3}$] and A=[Atf3]. Here all non-zero initial conditions are given by $m = m_0$, $E = E_0$, $T_E = T_{E0}$ and $T_A = T_{A0}$ at $t = 0$.

Conservation of T_E and T_A means

$$T_E + T_{EP} + S = T_{E0}, \tag{25}$$

and

$$T_A + T_{AP} = T_{A0}, \tag{26}$$

which along with solving for m_P as detailed in (S6) gives the reduced system of equations

$$m_P(t) = m_0\left(1 - e^{-k_1 e_T t}\right), \quad (27)$$

$$\frac{dE}{dt} = -k_2 m_P E, \quad (28)$$

$$\frac{dE_P}{dt} = k_2 m_P E - d_9 E_P, \quad (29)$$

$$\frac{dT_{EP}}{dt} = k_3 E_P (T_{E0} - T_{EP} - S) - k_{-3} E_P T_{EP} - k_7 T_{EP} A + k_{-7} S - d_{p1} T_{EP}, \quad (30)$$

$$\frac{dT_{AP}}{dt} = k_5 E_P (T_{A0} - T_{AP}) - k_{-5} T_{AP} E_P - d_{p2} T_{AP}, \quad (31)$$

$$\frac{dM_E}{dt} = k_4 T_{EP} - d_1 M_E, \quad (32)$$

$$\frac{dM_A}{dt} = k_6 T_{AP} - d_2 M_A, \quad (33)$$

$$\frac{dA}{dt} = k_8 M_A - k_7 T_{EP} A + k_{-7} S - d_3 A, \quad (34)$$

$$\frac{dS}{dt} = k_7 T_{EP} A - k_{-7} S, \quad (35)$$

with the initial conditions

$$E = E_0, \quad E_P = 0, \quad T_{EP} = 0, \quad T_{AP} = 0 \quad M_E = M_{E0}, \quad M_A = 0, \\ A = 0 \quad \text{and} \quad S = 0. \quad (36)$$

S1.2.1 Parameterisation, model solutions and sensitivity analysis

Model parameters are summarised in Table S1 in Text S1. The revision of our original model (S6) requires knowledge of 8 further parameters. These are: (i) the forward and reverse rates of bound transcription phosphorylation for Egr1 DNA and Atf3 DNA (k_3, k_{-3}, k_5, k_{-5}), respectively; (ii) the reverse rate of Atf3 binding to the phosphorylated transcription factor on Egr1 (this was excluded in our original model for reasons of simplification); (iii) the rate of ERK-P de-phosphorylation (d_9); and (iv) de-phosphorylation rates for the phosphorylated transcription factors bound to Egr1 and Atf3.

Given the qualitatitve nature of the data available to fit these new parameters (Figures 1D, 1F and 1G) a fit-by-eye was determined as the best method for fitting the mathematical model to the experimental data. Having determined these values a local sensitivity analysis, whereby each parameter was varied 2 orders of magnitude above and below their initially estimated value (see Table S1 in Text S1), was undertaken in order to evaluate how good a prediction each parameter estimate was. This was likewise undertaken for all the remaining models.

The forward and reverse rates of Egr1 and Atf3 bound transcription factor by ERK-P (k_3 and k_5) were obtained by fitting T_{EP} and T_{AP} to the data in Figures 1F and 1G,

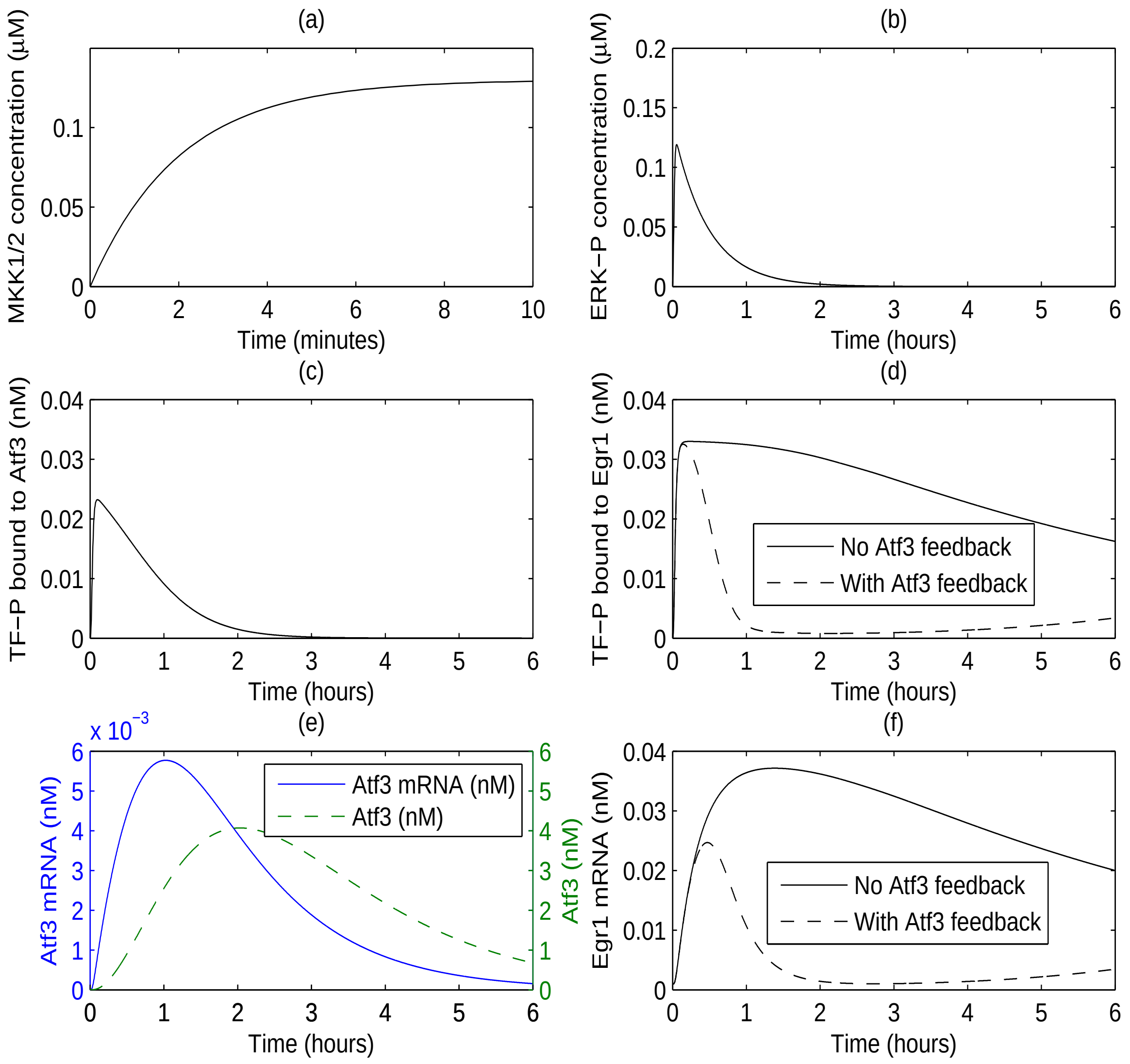

Figure S2: Revised original model: The predicted concentration of: (a) phosphorylated MKK1/2; (b) phosphorylated ERK; (c) T_{FP} bound to Atf3 DNA; (d) T_{FP} bound to Egr1 DNA; (e) Atf3 mRNA and Atf3 protein; and (f) Egr1 mRNA in the presence and absence of Atf3.

respectively. Estimates were then made of the reverse rates (k_{-3} and k_{-5}) as shown in Table S1 in Text S1. ERK-P de-phosphorylation (d_9) was obtained by making a good fit-by-eye between the data shown in Figure 1D. An initial estimate of 1.00 $\times 10^{-3}$ s^{-1} was made for k_{-7}, the rate of reverse association of Atf3 for Egr1 DNA, however, this value greatly decreased the levels of Egr1 mRNA below those experimentally observed (see Figure 1B). Utilising our sensitivity analysis we determined a value of 1.00 $\times 10^{-4}$ s^{-1} gave a much better fit to the model. The dephosphorylation rates of the Egr1 and Atf3 bound transcription factors ($\text{TF-P}_{\text{Egr1}}$ and $\text{TF-P}_{\text{Atf3}}$) were determined by fitting the model with data shown in Figures 1F and 1G. Finally, a best fit for the Egr1 data was obtained by increasing the rate of both Atf3 and Egr1 transcription 5-fold in comparison to that originally stated in (S6). This increase is feasible given the earlier work used the lower bounded estimate of transcription for both genes.

We also chose to investigate variation of all the remaining parameter values, each of which had been informed from experimental data within the literature as detailed in Table S1 in Text S1. These results revealed no unexpected behaviour. For instance decreasing the rate of Egr1 transcription meant not enough Egr1 mRNA was produced to be discernable; increasing Egr1 transcription did not affect the initially observed results. Decreasing the transcription of Atf3 reduces the amount of Atf3 produced, which in turn increases the amount of Egr1 mRNA (via the negative feedback relationship) such that it no longer fits our experimental data. Decreasing and increasing the rate of Atf3 translation had a similar effect. Likewise increasing/decreasing Atf3 mRNA degradation and likewise Atf3 led to more/less Egr1 mRNA being produced.

Solutions to the revised model are shown in Figure S2 in Text S1.

S2 Model Extension 1 - Atf3 protein inhibits Atf3 transcription

Here we consider Pathway 1 in Figure S1 in Text S1 where Atf3 protein binds to Atf3 DNA to inhibit its own transcription

$$\text{Atf3} + \text{DNA}_{\text{Atf3}} \underset{k_{-11}}{\overset{k_{11}}{\rightleftharpoons}} \text{Atf3} \cdot \text{DNA}_{\text{Atf3}}, \tag{37}$$

which is in competition with the binding of the transcription factor TF_{Atf3} which is required for Atf3 transcription

$$\text{TF}_{\text{Atf3}} + \text{DNA}_{\text{Atf3}} \underset{\lambda_{-1}}{\overset{\lambda_{1}}{\rightleftharpoons}} \text{TF}_{\text{Atf3}} \cdot \text{DNA}_{\text{Atf3}}, \tag{38}$$

and which is subsequently phosphorylated by ERK-P

$$\text{TF}_{\text{Atf3}} \cdot \text{DNA}_{\text{Atf3}} + \text{ERK-P} \underset{k_{-5}}{\overset{k_{5}}{\rightleftharpoons}} \text{TF-P}_{\text{Atf3}} \cdot \text{DNA}_{\text{Atf3}} + \text{ERK-P}. \tag{39}$$

Parameter	Definition	Value	Source
m_0	Total MKK.	130nM	(S6)
E_0	Total ERK.	130nM	(S6)
M_{E0}	Initial Egr1 mRNA concentration.	1pM	(S6)
D_{e0}	Egr1 DNA concentration.	33.2pM	(S6)
$k_1 e_t$	Rate of MKK activation by ET-1.	8.30×10^{-3} s^{-1}	(S6)
k_2	Rate of ERK activation by MKK.	1.28×10^{5} (Ms)$^{-1}$	(S6)
k_3	Rate of ERK-P phosphorylation of $\mathrm{TF}_{\mathrm{Egr1}}\cdot\mathrm{DNA}_{\mathrm{Egr1}}$.	1.00×10^{5} (Ms)$^{-1}$	This study.
k_{-3}	Rate of ERK-P reverse phosphorylation of $\mathrm{TF}_{\mathrm{Egr1}}\cdot\mathrm{DNA}_{\mathrm{Egr1}}$.	1.00×10^{2} (Ms)$^{-1}$	This study.
k_4	Egr1 mRNA transcription rate.	1.04×10^{-3} s^{-1}	This study.
k_6	Atf3 mRNA transcription rate.	1.55×10^{-4} s^{-1}	This study.
k_7	Rate of Atf3 suppression of Egr1.	1.00×10^{6} (Ms)$^{-1}$	This study.
k_{-7}	Reverse rate of Atf3 suppression of Egr1.	1.00×10^{-4} s^{-1}	This study.
k_8	Atf3 translation rate.	0.25 s^{-1}	(S6)
k_5	Rate of ERK-P phosphorylation of of $\mathrm{TF}_{\mathrm{Atf3}}\cdot\mathrm{DNA}_{\mathrm{Atf3}}$.	1.00×10^{5} (Ms)$^{-1}$	This study.
k_{-5}	Rate of ERK-P reverse phosphorylation	1.00×10^{2} (Ms)$^{-1}$	This study.
d_{p1}	Dephosphorylation rate of $\mathrm{TF}_{\mathrm{Egr1}}$.	4.72×10^{-5} s^{-1}	This study.
d_{p2}	Dephosphorylation rate of $\mathrm{TF}_{\mathrm{Atf3}}$.	4.72×10^{-3} s^{-1}	This study.
d_1	Degradation rate of Atf3 mRNA.	8.89×10^{-4} s^{-1}	(S6)
d_2	Degradation rate of Egr1 mRNA.	2.36×10^{-4} s^{-1}	(S6)
d_3	Degradation rate of Atf3 protein.	2.36×10^{-4} s^{-1}	(S6)
d_9	Rate of ERK-P de-phosphorylation.	5.90×10^{-4} s^{-1}	This study.

Table S1: Model parameter values for the Revised Model of Atf3 protein suppressing Egr1 mRNA expression.

The phosphorylated transcription factor can undergo de-phosphorylation such that

$$\text{TF-P}_{\text{Atf3}} \cdot \text{DNA}_{\text{Atf3}} \overset{d_{p2}}{\to} \text{TF}_{\text{Atf3}} \cdot \text{DNA}_{\text{Atf3}}. \tag{40}$$

This system of reactions leads to the following additional (for TF_{Atf3}, DNA_{Atf3} and Atf3$\cdot\text{DNA}_{\text{Atf3}}$) and revised (for Atf3 and $\text{TF}_{\text{Atf3}}\cdot\text{DNA}_{\text{Atf3}}$) ODEs

$$\begin{aligned}
\frac{dF}{dt} &= -\lambda_1 F D_A + \lambda_{-1} T_A, & (41)\\
\frac{dT_A}{dt} &= \lambda_1 F D_A - \lambda_{-1} T_A - k_5 T_A E_P + k_{-5} T_{AP} E_P + d_{p2} T_{AP}, & (42)\\
\frac{dT_{AP}}{dt} &= k_5 T_A E_P - k_{-5} T_{AP} E_P - d_{p2} T_{AP}, & (43)\\
\frac{dA}{dt} &= -k_{11} A D_A + k_{-11} T_{AS} - k_7 T_{EP} A + k_{-7} S + k_8 M_A - d_3 A, & (44)\\
\frac{dD_A}{dt} &= -\lambda_1 F D_A + \lambda_{-1} T_A - k_{11} A D_A + k_{-11} T_{AS}, & (45)\\
\frac{dT_{AS}}{dt} &= k_{11} A D_A - k_{-11} T_{AS}, & (46)
\end{aligned}$$

with

$$F = F_0 \quad \text{and} \quad D_A = D_{A0} \tag{47}$$

at $t = 0$ and all other initial conditions are zero. Here F=[TF_{Atf3}], D_A=[DNA_{Atf3}] and T_{AS}=[$\text{TF}_{\text{Atf3}}\cdot\text{DNA}_{\text{Atf3}}$].

Conservation of D_A (adding equations (42), (43), (45) and (46), integrating with respect to time and applying the initial conditions) leads to

$$D_A + T_A + T_{AP} + T_{AS} = D_{A0}. \tag{48}$$

A similar relationship holds for F

$$F + T_A + T_{AP} = F_0. \tag{49}$$

Assuming equation (46) is quasi-steady and utilising equation (48) leads to

$$k_{11} A(D_{A0} - T_A - T_{AP} - T_{AS}) - k_{-11} T_{AS} \simeq 0. \tag{50}$$

which on re-arranging gives

$$T_{AS} \simeq \frac{A}{(A + K_{11})}(D_{A0} - T_A - T_{AP}), \tag{51}$$

where $K_{11} = k_{-11}/k_{11}$.

This leads to the revised system of equations

$$m_P(t) = m_0\left(1 - e^{-k_1 e_T t}\right), \tag{52}$$

$$\frac{dE}{dt} = -k_2 m_P E, \tag{53}$$

$$\frac{dE_P}{dt} = k_2 m_P E - d_9 E_P, \tag{54}$$

$$\frac{dT_{EP}}{dt} = k_3 E_P (T_{E0} - T_{EP} - S) - k_{-3} E_P T_{EP} - k_7 T_{EP} A + k_{-7} S - d_{p1} T_{EP}, \tag{55}$$

$$\frac{dT_A}{dt} = \frac{\lambda_1 K_{11}}{A + K_{11}} (F_0 - T_A - T_{AP})(D_{A0} - T_A - T_{AP}) - \lambda_{-1} T_A - k_5 E_P T_A + k_{-5} T_{AP} E_P + d_{p2} T_{AP}, \tag{56}$$

$$\frac{dT_{AP}}{dt} = k_5 E_P T_A - k_{-5} T_{AP} E_P - d_{p2} T_{AP}, \tag{57}$$

$$\frac{dM_E}{dt} = k_4 T_{EP} - d_1 M_E, \tag{58}$$

$$\frac{dM_A}{dt} = k_6 T_{AP} - d_2 M_A, \tag{59}$$

$$\frac{dA}{dt} = k_8 M_A - k_7 T_{EP} A + k_{-7} S - d_3 A, \tag{60}$$

$$\frac{dS}{dt} = k_7 T_{EP} A - k_{-7} S, \tag{61}$$

with the initial conditions

$$E = E_0, \quad E_P = 0, \quad T_{EP} = 0, \quad T_A = 0, \quad T_{AP} = 0, \quad M_E = M_{E0}$$
$$M_A = 0, \quad A = 0, \quad \text{and} \quad S = 0.$$

S2.1 Parameterisation, model solutions and sensitivity analysis

This model requires three new parameters: (i) the association constant for which Atf3 protein associates with Atf3 DNA (K_{11}); and (ii) the forward and reverse rates of the Atf3 transcription factor for Atf3 DNA. These were informed as follows. K_{11} was assumed to have an initial value of $K_{11} = 0.1$nM, a value in line with that previously found in (S6). This value was varied in order to understand how Atf3 association for its DNA affected the Atf3 mRNA and Atf3 concentration profiles as discussed in the main text. The ratio of λ_{-1}/λ_1 was assumed to have a similar value to K_{11}, but a slow rate of reversal, such that $\lambda_{-1} = 1 \times 10^{-4}/s$ was chosen. This leads to a value of $\lambda_1 = 1 \times 10^5 (Ms)^{-1}$. Variations to λ_1 and λ_{-1} (informed by a sensitivity analysis) showed that decreasing this ratio 2-fold had no effect on the results shown in Figure S3 in Text S1. Increasing the ratio 2-fold decreased the amount of bound Atf3 transcription factor and thus the amount of Atf3, which increased the amount of Egr1 which does agree with the experimental data shown in Figure 1B. The effect of increasing the rate of association of Atf3 for Atf3 DNA (K_{11}) is shown in Figures 1B and 1C of the main text.

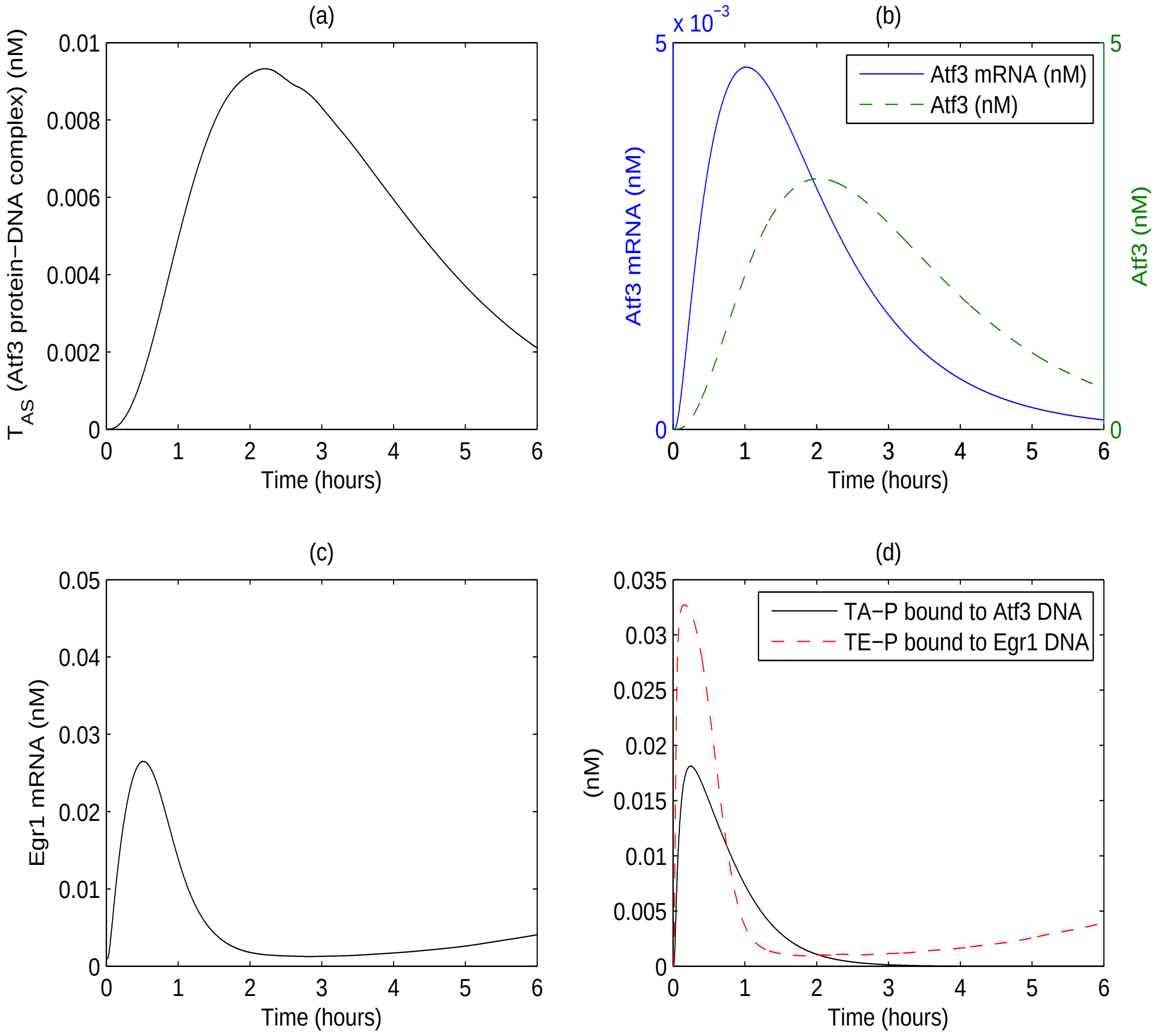

Figure S3: Pathway 1 - Atf3 protein inhibits Atf3 DNA transcription: The predicted concentration of: (a) the Atf3 bound to Atf3 DNA ; (b) Atf3 mRNA and Atf3 ; (c) Egr1 mRNA; and (d) the phosphorylated transcription factors bound to Atf3 and Egr1. The concentration of MKK1/2 and ERK-P are the same as those shown in Figures S2(a) and (b).

S3 Model Extension 2 - RSK-P inhibits Atf3 DNA transcription

Here Pathway 2 in Figure S1 in Text S1 acts and leads to RSK being phosphorylated by ERK-P

$$\text{ERK-P} + \text{RSK} \overset{k_9}{\rightarrow} \text{ERK-P} + \text{RSK-P}. \tag{62}$$

Two transcription factors then compete for sites on the Atf3 DNA, that which binds to Atf3 and is phosphorylated by ERK-P (as per equations (38) and (39)), and RTF

$$\text{RTF} + \text{DNA}_{\text{Atf3}} \underset{\lambda_{-2}}{\overset{\lambda_2}{\rightleftharpoons}} \text{RTF} \cdot \text{DNA}_{\text{Atf3}}, \tag{63}$$

which is subsequently phosphorylated by RSK-P

$$\text{RTF} \cdot \text{DNA}_{\text{Atf3}} + \text{RSK-P} \underset{k_{-10}}{\overset{k_{10}}{\rightleftharpoons}} \text{RTF-P} \cdot \text{DNA}_{\text{Atf3}} + \text{RSK-P}. \tag{64}$$

The additional and revised governing ODEs for these reactions are given by

$$\frac{dR}{dt} = -k_9 E_p R, \tag{65}$$

$$\frac{dR_P}{dt} = k_9 E_P R - d_{10} R_P, \tag{66}$$

$$\frac{dD_A}{dt} = -\lambda_1 F D_A + \lambda_{-1} T_A - \lambda_2 F_R D_A + \lambda_{-2} T_R, \tag{67}$$

$$\frac{dF_R}{dt} = -\lambda_2 F_R D_A + \lambda_{-2} T_R, \tag{68}$$

$$\frac{dT_R}{dt} = \lambda_2 F_R D_A - \lambda_{-2} T_R - k_{10} T_R R_P + k_{-10} T_{RP} R_P + d_{p5} T_{RP}, \tag{69}$$

$$\frac{dT_{RP}}{dt} = k_{10} T_R R_P - k_{-10} T_{RP} R_P - d_{p5} T_{RP}, \tag{70}$$

where R=[RSK], R_P=[RSK-P], F_R=[RTF], T_R=[RTF $\cdot$ DNA$_{\text{Atf3}}$] and T_{RP}=[RTF-P $\cdot$ DNA$_{\text{Atf3}}$] and the initial conditions are defined by

$$R = R_0, \quad R_P = 0, \quad D_A = D_{A0}, \quad F_R = F_{R0}, \quad T_R = 0 \quad \text{and} \quad T_{RP} = 0. \tag{71}$$

Conservation of Atf3 DNA holds from the addition of equations (42), (43), (67), (69) and (70) such that

$$D_A + T_A + T_R + T_{AP} + T_{RP} = D_{A0}, \tag{72}$$

and likewise that of RTF

$$F_R + T_R + T_{RP} = F_{R0}. \tag{73}$$

We also note that equation (49) still holds.

Bringing these results together, the revised systems of governing equations is given by

$$
\begin{aligned}
m_P(t) &= m_0\left(1 - e^{-k_1 e_T t}\right), && (74)\\
\frac{dE}{dt} &= -k_2 m_P E, && (75)\\
\frac{dE_P}{dt} &= k_2 m_P E - d_9 E_P, && (76)\\
\frac{dR}{dt} &= -k_9 R E_P, && (77)\\
\frac{dR_P}{dt} &= k_9 R E_P - d_{10} R_P, && (78)\\
\frac{dT_{EP}}{dt} &= k_3 E_P (T_{E0} - T_{EP} - S) - k_{-3} E_P T_{EP} - k_7 A T_{EP} + k_{-7} S - d_{p1} T_{EP}, && (79)\\
\frac{dT_A}{dt} &= \lambda_1 (D_{A0} - T_A - T_R - T_{AP} - T_{RP})(F_0 - T_A - T_{AP}) - \lambda_{-1} T_A \\
&\quad - k_5 E_P T_A + k_{-5} T_{AP} E_P + d_{p2} T_{AP}, && (80)\\
\frac{dT_R}{dt} &= \lambda_2 (D_{A0} - T_A - T_R - T_{AP} - T_{RP})(F_{R0} - T_R - T_{RP}) - \lambda_{-2} T_R \\
&\quad - k_{10} R_P T_R + k_{-10} T_{RP} R_P + d_{p5} T_{RP}, && (81)\\
\frac{dT_{AP}}{dt} &= k_5 E_P T_A - k_{-5} T_{AP} E_P - d_{p2} T_{AP}, && (82)\\
\frac{dT_{RP}}{dt} &= k_{10} R_P T_R - k_{-10} T_{RP} R_P - d_{p5} T_{RP}, && (83)\\
\frac{dM_E}{dt} &= k_4 T_{EP} - d_1 M_E, && (84)\\
\frac{dM_A}{dt} &= k_6 T_{AP} - d_2 M_A, && (85)\\
\frac{dA}{dt} &= k_8 M_A - k_7 T_{EP} A + k_{-7} S - d_3 A, && (86)\\
\frac{dS}{dt} &= k_7 T_{EP} A - k_{-7} S, && (87)
\end{aligned}
$$

with the initial conditions

$$
\begin{aligned}
&E = E_0, \quad E_P = 0, \quad R = R_0, \quad R_P = 0, \quad T_{EP} = 0, \quad T_A = 0, \quad T_R = 0, \quad T_{AP} = 0 \\
&T_{RP} = 0, \quad M_E = M_{E0}, \quad M_A = 0, \quad A = 0 \quad \text{and} \quad S = 0. \qquad (88)
\end{aligned}
$$

S3.1 Parameterisation, model solutions and sensitvity analysis

This model extension leads to the new parameters R_0 (initial concentration of RSK), k_9 (rate of RSK phosphorylation by ERK-P), d_{10} (rate of RSK-P de-phosphorylation),

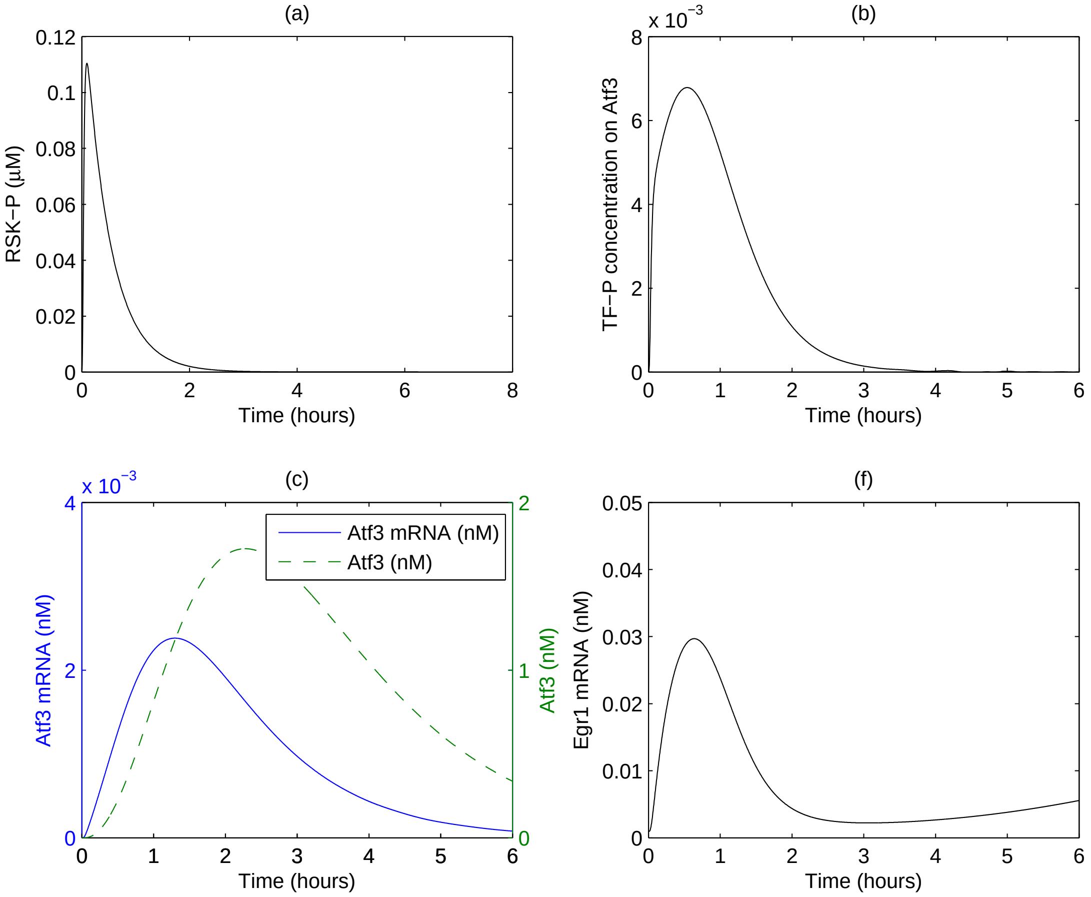

Figure S4: Pathway 2 - RSK-P inhibits Atf3 transcription: The predicted concentration of: (a) phosphorylated RSK; (b) phosphorylated transcription factor bound to Atf3 DNA; (c) Atf3 mRNA and Atf3; and (d) Egr1 mRNA. The concentration of MKK1/2 and ERK-P are the same as those shown in Figures S2(a) and (b).

λ_2 and λ_{-2} (the rates of association and disassociation of RTF for Atf3 DNA) and k_{10} and k_{-10} (the rates of phosphorylation and de-phosphorylation of RTF bound to Atf3 DNA). A fit-by-eye to the RSK data shown in Figure 3C yielded $k_9 = 1 \times 10^5$ (Ms)$^{-1}$ and $d_{10} = 5.9 \times 10^{-4}$ s^{-1}. We assume that λ_2=λ_1 and λ_{-2}=λ_{-1}, $k_{10} = k_5$, $k_{-10} = k_{-5}$ and $R_0 = E_0$ (the total amount of RSK is assumed equal to that of ERK).

Solutions to the model equations are shown in Figure S4 in Text S1. The effect of varying the competition between TF and RTF for Atf3 DNA on Atf3 mRNA, Atf3 protein and Egr1 mRNA levels (sensitivity analysis) are discussed in the main text.

S4 Model extension 3 - ITF suppresses Atf3 transcription

In this case Pathway 3 in Figure S1 in Text S1 acts and leads to ITF protein being transcribed by RSK-P. This pathway begins by RSK-P phosphorylating the transcription factor bound ITF DNA such that

$$\mathrm{TF_{ITF}} \cdot \mathrm{DNA_{ITF}} + \text{RSK-P} \quad \underset{k_{-12}}{\overset{k_{12}}{\rightleftharpoons}} \quad \text{TF-P}_{\mathrm{ITF}} \cdot \mathrm{DNA_{ITF}} + \text{RSK-P}, \tag{89}$$

which subsequently produces ITF mRNA

$$\text{TF-P}_{\mathrm{ITF}} \cdot \mathrm{DNA_{ITF}} \overset{k_{13}}{\rightarrow} \mathrm{mRNA_{ITF}}, \tag{90}$$

or de-phosphorylates

$$\text{TF-P}_{\mathrm{ITF}} \cdot \mathrm{DNA_{ITF}} \overset{d_{p3}}{\rightarrow} \mathrm{TF_{ITF}} \cdot \mathrm{DNA_{ITF}}. \tag{91}$$

The ITF mRNA is subsequently translated to ITF protein

$$\mathrm{mRNA_{ITF}} \overset{k_{14}}{\rightarrow} \mathrm{ITF}, \tag{92}$$

whereby ITF protein subsequently binds to Atf3 DNA to inhibit transcription

$$\mathrm{ITF} + \mathrm{DNA_{Atf3}} \quad \underset{k_{-15}}{\overset{k_{15}}{\rightleftharpoons}} \quad \mathrm{ITF} \cdot \mathrm{DNA_{Atf3}}. \tag{93}$$

Finally the ITF mRNA and protein each degrade

$$\mathrm{mRNA_{ITF}} \overset{d_4}{\rightarrow} \phi \quad \text{and} \quad \mathrm{ITF} \overset{d_5}{\rightarrow} \phi. \tag{94}$$

Applying the law of mass action to this series of reactions leads to

$$\frac{dT_I}{dt} = -k_{12}T_IR_P + k_{-12}T_{IP}R_P + d_{p3}T_{IP}, \tag{95}$$

$$\frac{dT_{IP}}{dt} = k_{12}T_IR_P - k_{-12}T_{IP}R_P - d_{p3}T_{IP}, \tag{96}$$

$$\frac{dM_I}{dt} = k_{13}T_{IP} - d_4M_I, \tag{97}$$

$$\frac{dI}{dt} = k_{14}M_I - k_{15}ID_A + k_{-15}T_{IA} - d_5I, \tag{98}$$

$$\frac{dD_A}{dt} = -\lambda_1FD_A + \lambda_{-1}T_A - k_{15}ID_A + k_{-15}T_{IA}, \tag{99}$$

$$\frac{dF}{dt} = -\lambda_1FD_A + \lambda_{-1}T_A, \tag{100}$$

$$\frac{dT_A}{dt} = \lambda_1FD_A - \lambda_{-1}T_A - k_5T_AE_P + k_{-5}T_{AP}E_P + d_{p2}T_{AP}, \tag{101}$$

$$\frac{dT_{AP}}{dt} = k_5T_AE_P - k_{-5}T_{AP}E_P - d_{p2}T_{AP}, \tag{102}$$

$$\frac{dT_{IA}}{dt} = k_{15}ID_A - k_{-15}T_{IA}, \tag{103}$$

where T_I=[$\text{TF}_{\text{ITF}}\cdot\text{DNA}_{\text{ITF}}$], T_{IP}=[$\text{TF-P}_{\text{ITF}}\cdot\text{DNA}_{\text{ITF}}$], M_I=[mRNA_{ITF}], I=[ITF], T_{IA}=[$\text{RSK}\cdot\text{DNA}_{\text{ITF}}$] and the initial conditions are given by

$$T_I = T_{I0}, \quad T_{IP} = 0, \quad M_I = 0, \quad I = 0, \quad D_A = D_{A0}, \quad F = F_0, \quad T_A = 0, \quad T_{AP} = 0 \quad \text{and} \quad T_{IA} = 0. \tag{104}$$

As with the previous pathways we first observe that addition of equations (99), (101), (102) and (103) leads to conservation of Atf3 DNA such that

$$D_A + T_A + T_{AP} + T_{IA} = D_{A0}. \tag{105}$$

Likewise conservation of the transcription factor bound to Atf3 DNA holds as defined by equation (49) and addition of equations (95) and (96) gives

$$T_I + T_{IP} = T_{I0}. \tag{106}$$

Assuming equation (103) is quasi-steady leads to

$$T_{IA} \simeq \frac{I}{I + K_{15}}(D_{A0} - T_A - T_{AP}). \tag{107}$$

Utilising these results leads to the simplified system of

$$m_P(t) = m_0\left(1 - e^{-k_1 e_T t}\right), \tag{108}$$

$$\frac{dE}{dt} = -k_2 m_P E, \tag{109}$$

$$\frac{dE_P}{dt} = k_2 m_P E - d_9 E_P, \tag{110}$$

$$\frac{dR}{dt} = -k_9 R E_P, \tag{111}$$

$$\frac{dR_P}{dt} = k_9 R E_P - d_{10} R_P, \tag{112}$$

$$\frac{dT_{IP}}{dt} = k_{12}(T_{I0} - T_{IP})R_P - k_{-12} T_{IP} R_P - d_{p3} T_{IP}, \tag{113}$$

$$\frac{dM_I}{dt} = k_{13} T_{IP} - d_4 M_I, \tag{114}$$

$$\frac{dI}{dt} = k_{14} M_I - d_5 I, \tag{115}$$

$$\frac{dT_{EP}}{dt} = k_3 E_P (T_{E0} - T_{EP} - S) - k_{-3} E_P T_{EP} - k_7 A T_{EP} + k_{-7} S - d_{p1} T_{EP}, \tag{116}$$

$$\begin{aligned}\frac{dT_A}{dt} = {} & \frac{\lambda_1 K_{15}}{I + K_{15}}(F_0 - T_A - T_{AP})(D_{A0} - T_A - T_{AP}) - \lambda_{-1} T_A \\ & - k_5 E_P T_A + k_{-5} T_{AP} E_P + d_{p2} T_{AP},\end{aligned} \tag{117}$$

$$\frac{dT_{AP}}{dt} = k_5 E_P T_A - k_{-5} T_{AP} E_P - d_{p2} T_{AP}, \tag{118}$$

$$\frac{dM_E}{dt} = k_4 T_{EP} - d_1 M_E, \tag{119}$$

$$\frac{dM_A}{dt} = k_6 T_{AP} - d_2 M_A, \tag{120}$$

$$\frac{dA}{dt} = k_8 M_A - k_7 T_{EP} A + k_{-7} S - d_3 A, \tag{121}$$

$$\frac{dS}{dt} = k_7 T_{EP} A - k_{-7} S, \tag{122}$$

with the initial conditions

$$\begin{aligned} & E = E_0, \quad E_P = 0, \quad R = R_0, \quad R_P = 0, \quad T_{IP} = 0, \quad M_I = 0, \quad I = 0, \quad T_{EP} = 0, \\ & T_A = 0, \quad T_{AP} = 0, \quad M_E = M_{E0}, \quad M_A = 0, \quad A = 0 \quad \text{and} \quad S = 0. \end{aligned} \tag{123}$$

S4.1 Parameterisation, model solutions and sensitivity analysis

We require six further parameters for this model: (i) the disassociation constant of ITF protein for Atf3 DNA (K_{15}), which we assume is equal to that of RSK-P for Atf3 DNA

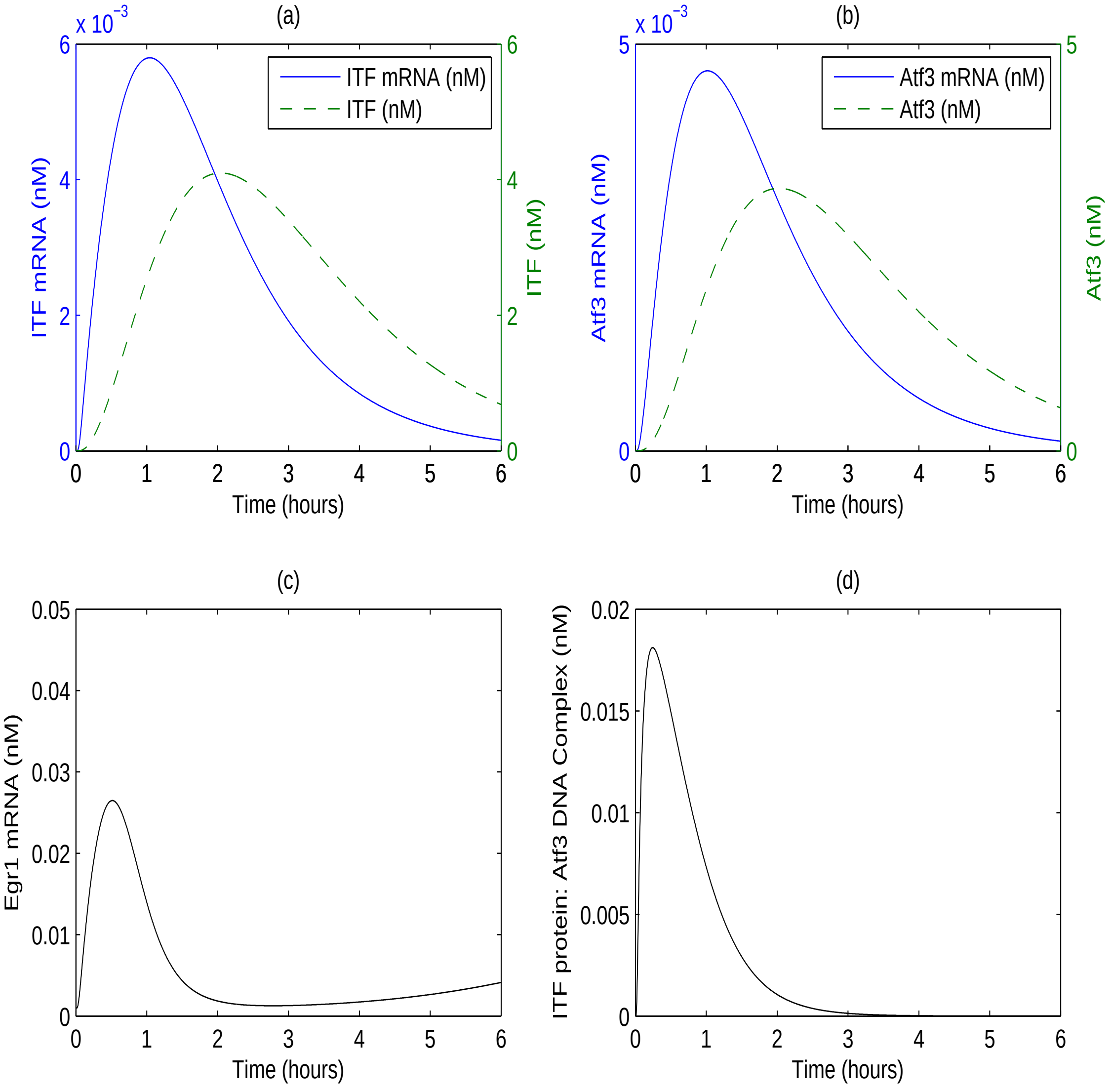

Figure S5: Model Extension 3 - ITF supresses Atf3 DNA transcription: The predicted concentration of: (a) ITF mRNA and ITF; (b) Atf3 mRNA and Atf3; (c) Egr1 mRNA; and (d) ITF protein bound to Atf3 DNA.

($K_{15} = K_{10}$); (ii) ITF transcription (k_{13}) which we assume equal to that of Atf3 transcription ($k_{13} = k_6$); (iii) translation rate of ITF protein (k_{14}) which we assume equal to that of Atf3 translation ($k_{14} = k_8$); (iv) bound ITF transcription factor dephosphorylation (d_{p3}), which we assume equal to that of Atf3 (d_{p3}=d_{p2}); (v) ITF mRNA degradation (d_4) which we assume equal to Atf3 mRNA degradation ($d_4 = d_2$); and (vi) ITF degradation (d_5) which we assume equal to that of Atf3 ($d_5 = d_3$).

Solutions to the model equations are shown in Figure S5 in Text S1 and are discussed in the main text. A sensitivity analysis of the model (methodology detailed in Section S1.2.1) revealed that increasing or decreasing K_{15} had no affect on the model solutions; the concentration in ITF is only briefly high enough to impeded Atf3 transcription (see Figure S5(d) in Text S1) and varying the disassociation rate 2 orders of magnitude up or down was not enough to affect the overall Atf3 response. Increasing/increasing k_{13} led to increases in ITF levels, but this do not affect the Atf3 mRNA or Atf3 profiles and subsequently the Egr1 mRNA profile remained the same. Varying the remaining parameters, k_{14}, d_4, d_5 and d_{p3} altered the amounts of ITF subsequently produced (mRNA and protein). Both of these variations were again not high to alter the Atf3 mRNA or Atf3 profiles and subsequently Egr1 mRNA given the transient nature of ITF binding to Atf3 DNA.

S5 Model Extension 4 - RSK-P driven microRNA expression increases Atf3 mRNA degradation and/or inhibits protein synthesis

Here we consider Pathway 4 in Figure S1 in Text S1 in which microRNA expression is facilitated by the phosphorylated RSK. The transcribed microRNA is then free to bind to Atf3 RNA whereby it forms an inactive complex, thus reducing the quantity of mRNA available for Atf3 translation.

At the start of this pathway

$$\text{ERK-P} + \text{RSK} \overset{k_9}{\to} \text{ERK-P} + \text{RSK-P}, \tag{124}$$

whereby RSK-P associates with the microDNA bound transcription factor to phosphorylate it

$$\text{TF} \cdot \text{miDNA} + \text{RSK-P} \quad \underset{k_{-16}}{\overset{k_{16}}{\rightleftharpoons}} \quad \text{TF-P} \cdot \text{miDNA} + \text{RSK-P}. \tag{125}$$

The phosphorylated transcription factor subsequently produces premiRNA

$$\text{TF-P} \cdot \text{miDNA} \quad \overset{k_{17}}{\to} \quad \text{premiRNA}, \tag{126}$$

or de-phosphorylates

$$\text{TF-P} \cdot \text{miDNA} \quad \stackrel{d_{p4}}{\rightarrow} \quad \text{TF} \cdot \text{miDNA}. \tag{127}$$

miRNA is produced from the premiRNA

$$\text{premiRNA} \stackrel{k_{18}}{\rightarrow} \text{miRNA} \tag{128}$$

and both subsequently degrade

$$\text{premiRNA} \stackrel{d_6}{\rightarrow} \phi \quad \text{and} \quad \text{miRNA} \stackrel{d_7}{\rightarrow} \phi. \tag{129}$$

Finally the miRNA can bind to the Atf3 RNA to form an inactive complex which inhibits Atf3 RNA translation

$$\text{miRNA} + \text{mRNA}_{\text{Atf3}} \quad \underset{k_{-19}}{\overset{k_{19}}{\rightleftharpoons}} \quad \text{miRNA} \cdot \text{mRNA}_{\text{Atf3}}, \tag{130}$$

and the complex can subsequently be degraded such that

$$\text{miRNA} \cdot \text{mRNA}_{\text{Atf3}} \stackrel{d_8}{\rightarrow} \phi. \tag{131}$$

The additional ODEs describing these reactions are given by

$$\frac{dT_m}{dt} = -k_{16} T_m R_P + k_{-16} T_{mP} R_P + d_{P4} T_{mP}, \tag{132}$$

$$\frac{dT_{mP}}{dt} = k_{16} T_m R_P - k_{-16} T_{mP} R_P - d_{P4} T_{mP}, \tag{133}$$

$$\frac{dm_P}{dt} = k_{17} T_{mP} - d_6 m_P, \tag{134}$$

$$\frac{dm_R}{dt} = k_{18} m_P - k_{19} m_R M_A + k_{-19} T_M - d_7 m_R, \tag{135}$$

$$\frac{dM_A}{dt} = k_6 T_A - k_{19} m_R M_A + k_{-19} T_M - d_2 M_A, \tag{136}$$

$$\frac{dT_M}{dt} = k_{19} m_R M_A - k_{-19} T_M - d_8 T_M, \tag{137}$$

where T_m=[TF·miDNA], T_{mP}=[TF-P·miDNA], m_P=[premiRNA], m_R=[miRNA], T_M=[miRNA·mRNA$_{\text{Atf3}}$], with the initial conditions

$$T_m = T_{m0}, \quad T_{mP} = 0, \quad m_P = 0, \quad m_R = 0, \quad M_A = 0 \quad \text{and} \quad T_M = 0. \tag{138}$$

From this system of equations we see that the quantity of miDNA transcription factor is conserved (summation of equations (132) and (133))

$$T_m + T_{mP} = T_{m0}. \tag{139}$$

This allows us to reduce the system of governing ODEs for Pathway 4 to

$$\begin{aligned}
m_P(t) &= m_0\left(1 - e^{-k_1 e_T t}\right), && (140)\\
\frac{dE}{dt} &= -k_2 m_P E, && (141)\\
\frac{dE_P}{dt} &= k_2 m_P E - d_9 E_P, && (142)\\
\frac{dR}{dt} &= -k_9 R E_P, && (143)\\
\frac{dR_P}{dt} &= k_9 R E_P - d_{10} R_P, && (144)\\
\frac{dT_{EP}}{dt} &= k_3 E_P (T_{E0} - T_{EP} - S) - k_{-3} E_P T_{EP} - k_7 T_{EP} A + k_{-7} S - d_{p1} T_{EP}, && (145)\\
\frac{dT_{AP}}{dt} &= k_5 E_P (T_{A0} - T_{AP}) - k_{-5} T_{AP} E_P - d_{p2} T_{AP}, && (146)\\
\frac{dM_E}{dt} &= k_4 T_{EP} - d_1 M_E, && (147)\\
\frac{dM_A}{dt} &= k_6 T_A P - k_{19} m_R M_A + k_{-19} T_M - d_2 M_A, && (148)\\
\frac{dA}{dt} &= k_8 M_A - k_7 T_E A + k_{-7} S - d_3 A, && (149)\\
\frac{dS}{dt} &= k_7 T_E A - k_{-7} S, && (150)\\
\frac{dT_{mP}}{dt} &= k_{16} (T_{m0} - T_{mP}) R_P - k_{-16} T_{mP} R_P - d_{P4} T_{mP}, && (151)\\
\frac{dm_P}{dt} &= k_{17} T_{mP} - d_6 m_P, && (152)\\
\frac{dm_R}{dt} &= k_{18} m_P - k_{19} m_R M_A + k_{-19} T_M - d_7 m_R, && (153)\\
\frac{dT_M}{dt} &= k_{19} m_R M_A - k_{-19} T_M - d_8 T_M, && (154)
\end{aligned}$$

with the initial conditions

$$\begin{gathered}
E = E_0, \quad E_P = 0, \quad R = R_0, \quad R_P = 0, \quad T_{EP} = 0, \quad T_{AP} = 0,\\
M_E = M_{E0}, \quad M_A = 0, \quad A = 0, \quad S = 0, \quad T_{mP} = 0, \quad m_P = 0,\\
m_R = 0 \quad \text{and} \quad T_M = 0.
\end{gathered}$$

S5.1 Parameterisation, model solutions and sensitivity analysis

This extension to the model introduces eight new parameters. A number of these are currently unknown experimentally and we hence proceed to inform them as follows.

k_{16}, k_{-16} - We assume the rate of phosphorylation for the transcription factor bound miDNA is equal to that of the transcription factors bound to Egr1 and Atf3 by ERK-P ($k_{16} = k_3$). The reverse rate of phosphorylation (k_{-16}) is assumed equal to k_{-3}.

k_{17} - We assume the rate of miDNA transcription is the same as that of Atf3 mRNA transcription, i.e. $k_{17} = k_6$.

k_{18} - The rate at which premiRNA is converted to miRNA is assumed to be 0.5/s (double the rate of Atf3 translation).

k_{19} - The rate at which miRNA associates with Atf3 mRNA is assumed to be 1×10^5 $(\text{Ms})^{-1}$ (equivalent to the rate we have set for Atf3 protein binding Egr1 DNA).

k_{-19} - The reverse rate at which miRNA associates with Atf3 mRNA is assumed to be 5×10^{-5}/s - the value that was found to give the best qualitative fit to the experimental data.

d_6, d_7 and d_8 - We assume the rate of decay of premiRNA (d_6) and miRNA (d_7) takes approximately 24 hours, which is line with recently published data (S5; 10). This leads to a decay rate of $d_6 = 8.02 \times 10^{-6} = d_7$. The rate of decay of the complex miRNA$\cdot$mRNA$_{\text{Atf3}}$ is assumed the same as that of Atf3 mRNA, i.e. $d_8 = d_3$.

d_{p4} - This is assumed equal to that of phosphorylated Atf3 bound transcription factor degradation ($d_{p4} = d_{p2}$).

The effect of introducing the miRNA process on the expression levels of Atf3 mRNA is shown in Figure S6 in Text S1 and discussed in further detail in the main text. A sensitivity analysis of the model parameters (see Section S1.2.1 for methodology) revealed that when k_{16} was increased/decreased the Atf3 mRNA, Atf3 and Egr1 profiles remained the same, but less/more Atf3 was produced. This is because increasing/decreasing RSK-P phosphosrylation of the transcription factor bound to the miDNA produces more/less miRNA which leads to less/more Atf3 mRNA and subsequently Atf3. However, this affect is not so large over the two orders of magnitude variation in k_{16} so as to dramatically affect the concentration of Atf3 mRNA, Atf3 and Egr1 mRNA. The same effect was observed when altering k_{-16}. It was found that varying k_{19} and k_{-19} had the same effect due to the immediate association between miRNA and Atf3 mRNA in increasing/decreasing levels of Atf3 mRNA.

Increasing k_{17} decreased the amount of Atf3 mRNA and thus Atf3 which subseqently led to an increase in Egr1 mRNA, such that it no longer matched the experimental data. A similar result was found when k_{17} was decreased meaning that the Atf3 mRNA profile no longer fitted the experimental data. In each case the Egr1 mRNA profile remained the same. Similar effects were observed when k_{18} was subsequently increased or decreased.

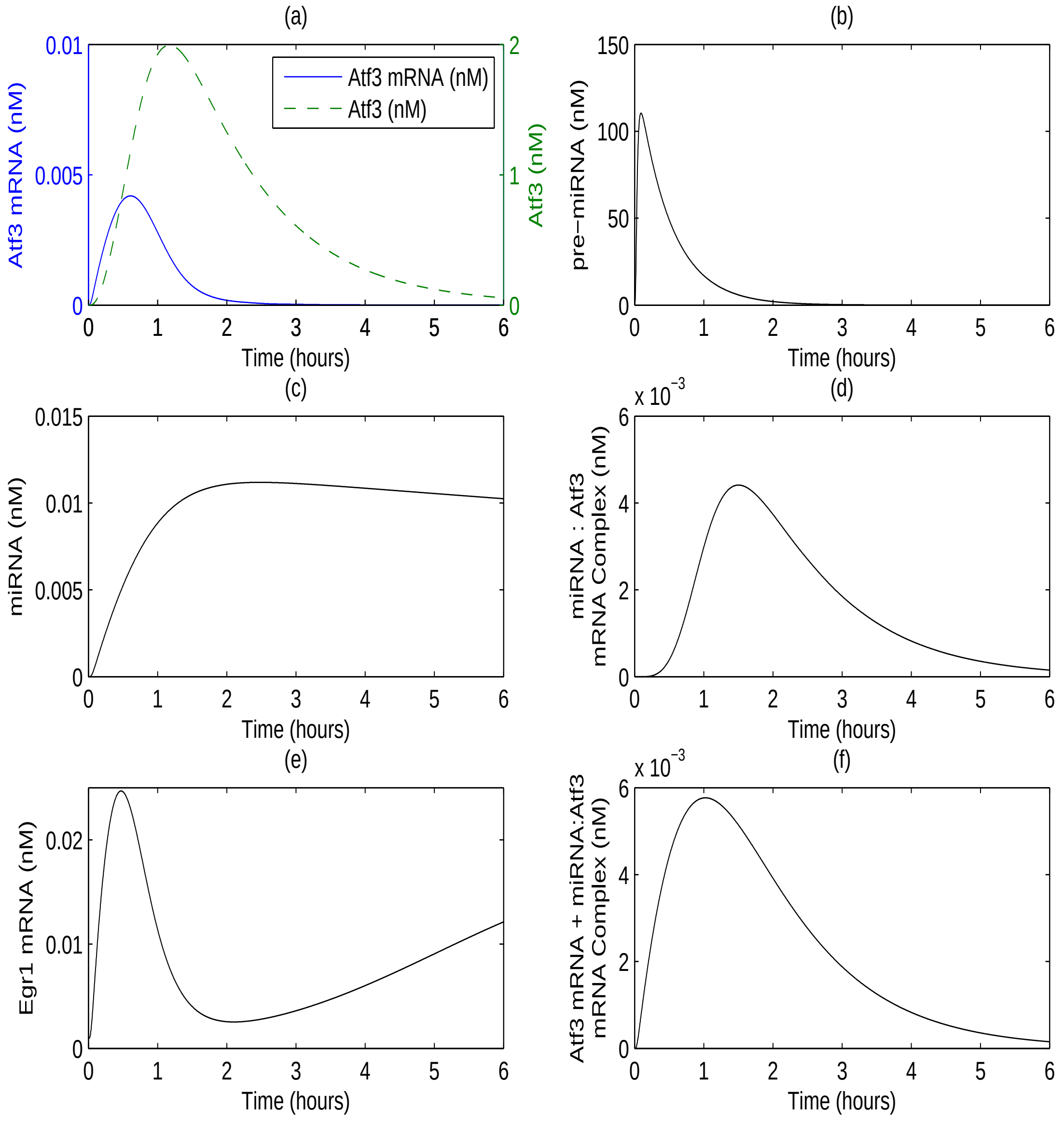

Figure S6: Pathway 4 - microRNA inhibits Atf3 mRNA: The predicted concentration of: (a) Atf3 mRNA and Atf3; (b) pre-miRNA; (c) miRNA; (d) miRNA·Atf3 mRNA complex; (e) Egr1 mRNA; and (f) total Af3 mRNA (free and bound).

When increasing and decreasing the rates of degradation d_6 and d_7 the solutions for Atf3 mRNA, Atf3 and Egr1 mRNA remained the same; the concentration of Atf3 mRNA and Atf3 either rose or fell, but not significantly. Increasing or decreasing d_8 had enough effect to allow improved fitting of the Atf3 mRNA solution with the experimental data (see main text for discussion). Increasing d_{p4} had more of an effect on the model - data fitting as it more strongly influences how much miRNA is produced early on in the signalling cascade; in this case the Atf3 mRNA profile no longer matches the experimental data as not enough miRNA was produced to reduce the Atf3 mRNA levels. However, the Egr1 mRNA remained the same. Decreasing d_{p4} had no appreciable effect on the solutions.

References

[S1] Ben-Ari, Y. et al. The life of an mRNA in space and time. *J. Cell Sci.* 123, 1761-1774 (2010).

[S2] Burkhard, K.A., Chen, F., & Shapiro, P. Quantitative analysis of ERK2 interactions with substrate proteins: roles for kinase docking domains and activity in determining binding affinity. *J. Biol. Chem.* 286, 2477-2485 (2011).

[S3] Cerbai, E., Pino, R., Sartiani, L., & Mugelli,A. Influence of postnatal-development on I(f) occurrence and properties in neonatal rat ventricular myocytes. *Cardiovasc. Res.* 42, 416-423 (1999).

[S4] Fujioka, A. et al. Dynamics of the Ras/ERK MAPK cascade as monitored by fluorescent probes. *J. Biol. Chem.* 281, 8917-8926 (2006).

[S5] Gantier, P.M. et al. Analysis of microRNA turnover in mammalian cells following *Dicer1* ablation. *Nucleic Acids Res.* 39 (13), 5692-5703 (2011).

[S6] Giraldo, A., Barrett, O.P.T., Tindall, M.J., Fuller, S.J., Amirak, E.A, Bhattacharya, B.S., Sugden, P.H. & Clerk, A. Feedback regulation by Atf3 in the endothelin-1-responsive transcriptome in cardiomyocytes: Egr1 is a principal Atf3 target. *Biochem. J.*, 444(2),343-55, (2012).

[S7] Maiuri, P. et al. Fast transcription rates of RNA polymerase II in human cells. *EMBO Rep.* (2011).

[S8] J.D. Murray, Mathematical Biology, Springer Verlag, 2nd ed., 1993.

[S9] Qin, X., Ahn, S., Speed, T.P., & Rubin, G.M. Global analyses of mRNA translational control during early *Drosophila* embryogenesis. *Genome Biol.* 8, R63 (2007).

[S10] Nazarov, P.V. et al. Interplay of microRNAs, transcription factors and target genes: linking dynamic expression changes to function. *Nucleic Acids Res.* doi:10.1093/nar/gks1471 (2013)

[S11] Satoh, H., Delbridge, L.M., Blatter, L.A., & Bers, D.M. Surface:volume relationship in cardiac myocytes studied with confocal microscopy and membrane capacitance measurements: species-dependence and developmental effects. *Biophys. J.* 70, 1494-1504 (1996).

[S12] Sugden, P.H. et al. Monophosphothreonyl extracellular signal-regulated kinases 1 and 2 (ERK1/2) are formed endogenously in intact cardiac myocytes and are enzymically active. *Cell. Signal.* 23, 468-477 (2011).

[S13] Tinoco, I., Jr. & Wen, J.D. Simulation and analysis of single-ribosome translation. *Phys. Biol.* 6, 025006 (2009).

[S14] Vassilenko, K.S., Alekhina, O.M., Dmitriev, S.E., Shatsky, I.N., & Spirin,A.S. Unidirectional constant rate motion of the ribosomal scanning particle during eukaryotic translation initiation. *Nucleic Acids Res.* 39, 5555-5567 (2011).

[S15] Wohlgemuth, I., Pohl, C., & Rodnina, M.V. Optimization of speed and accuracy of decoding in translation. *EMBO J.* 29, 3701-3709 (2010).
